# A new nutraceutical (Livogen Plus®) improves liver steatosis in adults with non-alcoholic fatty liver disease

**DOI:** 10.1186/s12967-022-03579-1

**Published:** 2022-08-19

**Authors:** Yvelise Ferro, Roberta Pujia, Elisa Mazza, Lidia Lascala, Oscar Lodari, Samantha Maurotti, Arturo Pujia, Tiziana Montalcini

**Affiliations:** 1 Department of Medical and Surgical Science, University Magna Græcia, 88100 Catanzaro, Italy; 2 Department of Clinical and Experimental Medicine, University Magna Greæcia, Catanzaro, Italy; 3 Research Center for the Prevention and Treatment of Metabolic Diseases (CR METDIS), University Magna Græcia, 88100 Catanzaro, Italy

**Keywords:** Nutraceutical, Fatty liver disease, Bioactive components, Hepatic steatosis

## Abstract

**Background:**

Currently, there is no approved medication for non-alcoholic fatty liver disease management. Pre-clinical and clinical studies showed that several bioactive molecules in plants or foods (i.e., curcumin complex, bergamot polyphenol fraction, artichoke leaf extract, black seed oil, concentrate fish oil, picroliv root, glutathione, *S*-adenosyl-l-methionine and other natural ingredients) have been associated with improved fatty liver disease. Starting from these evidences, our purpose was to evaluate the effects of a novel combination of abovementioned nutraceuticals as a treatment for adults with fatty liver disease.

**Methods:**

A total of 140 participants with liver steatosis were enrolled in a randomized, double-blind, placebo controlled clinical trial. The intervention group received six softgel capsules daily of a nutraceutical (namely Livogen Plus®) containing a combination of natural bioactive components for 12 weeks. The control group received six softgel capsules daily of a placebo containing maltodextrin for 12 weeks. The primary outcome measure was the change in liver fat content (CAP score). CAP score, by transient elastography, serum glucose, lipids, transaminases, and cytokines were measured at baseline and after intervention.

**Results:**

After adjustment for confounding variables (i.e., CAP score and triglyceride at baseline, and changes of serum γGT, and vegetable and animal proteins, cholesterol intake at the follow-up), we found a greater CAP score reduction in the nutraceutical group rather than placebo (− 34 ± 5 dB/m vs. − 20 ± 5 dB/m, respectively; p = 0.045). The CAP score reduction (%) was even greater in those with aged 60 or less, low baseline HDL-C, AST reduction as well as in men.

**Conclusion:**

Our results showed that a new combination of bioactive molecules as nutraceutical was safe and effective in reducing liver fat content over 12 weeks in individuals with hepatic steatosis.

*Trial registration* ISRCTN, ISRCTN70887063. Registered 03 August 2021—retrospectively registered, https://doi.org/10.1186/ISRCTN70887063

**Supplementary Information:**

The online version contains supplementary material available at 10.1186/s12967-022-03579-1.

## Background

Non-alcoholic fatty liver disease (NAFLD) is the most frequent chronic liver disorder worldwide, with an overall prevalence of approximately 25% of the adult population [1]. NAFLD is the hepatic component of a cluster of diseases that are associated with metabolic dysfunction, such as obesity, dyslipidemia, insulin resistance (IR) and/or type 2 diabetes (T2D) [1–3]. Fatty liver disease could progress in non-alcoholic steatohepatitis (NASH), severe liver fibrosis, cirrhosis and hepatocellular cancer [4, 5]. Total costs of NAFLD care independent of its metabolic comorbidities are very high [6]. Importantly, economic and health burden of fatty liver disease will probably increase during the coming decades [7, 8]. Weight loss and changing lifestyle behaviours are the first therapeutic approach to prevent NAFLD and its progression [9, 10]. However, most people with fatty liver disease do not adhere to this effective treatment approach. Nowadays, there are no approved therapeutic drugs available on the market able to reverse effectively NAFLD or slow its progression. Many natural bioactive components isolated from fruits, vegetables, and fish or produced by microorganisms could be promising agents capable of reversing NAFLD [11, 12]. In particular, some studies suggested that ω-3 polyunsaturated fatty acids (PUFAs) [12, 13]; curcumin [12, 14]; bergamot polyphenol fraction (BPF) [15, 16]; artichoke leaf extract [15–18]; black seed oil of *Nigella sativa* [19]; and the standardised fraction “Picroliv” of the root of *Picrorhiza kurroa* [20, 21] have anti-oxidant, anti-inflammatory, hypolipidemic and hypoglycemic proprieties [22, 23]. Moreover, they can also reduce hepatic steatosis and other liver injury, both in preclinical and clinical studies [12–21]. Many other natural compound are reported to have beneficial effects on NAFLD, including indole-3-carbinol (I3C) found in cruciferous vegetables [24–26]; silymarin [12, 27, 28] and silybin [29, 30] isolated from milk thistle; luteolin found in fruits, vegetables, and natural herbs [31, 32] astaxanthin produced by microalgae [33] and many others [34–40]. Besides the bioactive components present in the diet, also endogenous substances have shown potential therapeutic effects on the liver. Glutathione (GHS) is the major endogenous hepato-protective agent, and, *S*-adenosyl-l-methionine (SAMe), its precursor, has an important role in the prevention of oxidative stress [21, 41]. Administration of GHS and SAMe has been evaluated in the prevention and treatment of a variety of liver injuries [21, 42, 43].

According to the evidences of the positive effects of each aforementioned molecules on fatty liver disease, it is conceivable that the combination of several natural ingredients, which have different proprieties, could represent a new tool for treating NAFLD.

In this study, our aim was to assess the effect of a novel combination of nutraceuticals (i.e., curcumin complex, ω-3 PUFAs, BPF, artichoke leaf extract, black seed oil, pricoliv, GHS, SAMe and other natural ingredients) as a treatment for adults with liver steatosis. We also investigated the effects of the present nutraceuticals combination on the change of the intracellular lipid content in a cellular model of NAFLD.

## Methods

### Human study

#### Subjects

A sample of adult subjects who had previously been screened for the possible presence of NAFLD by Transient Vibration-Controlled Elastography (VCTE) (Fibroscan) were enrolled in a randomized, double-blind, placebo-controlled clinical study (RCT). We enrolled one hundred forty individuals with hepatic steatosis, aged between 30 and 75 attending the Clinical Nutrition Unit of the “Mater Domini” Azienda University Hospital in Catanzaro, Italy, (study duration between July 12, 2021 and December 23, 2021).

The RCT protocol was approved by the Local Ethics Committee in the Calabria Region—Central Area (330/2020/CE approved October 22, 2020), and registered at International Clinical Trials Registry Platform (ICTRP) with the identifier ISRCTN70887063. According to the RCT’s protocol, we excluded participants with clinical and laboratory signs of chronic hepatitis B and/or C virus infection or intolerance to nutraceutical components and subjects affected by diabetes. We also excluded subjects with autoimmune or cholestatic liver disease, liver cirrhosis, gastroesophageal reflux, nephrotic syndrome, pregnancy, chronic renal failure, cancer, and those taking methotrexate, corticosteroids, amiodarone, antiretroviral agents, valproate, tamoxifen, nutraceuticals, supplements or functional foods as confirmed by their medical records. We also excluded who started lipid-lowering therapy with < 1 month before study start. Furthermore, individuals with previous and actual alcohol abuse (> 20 g of alcohol per day) were excluded [15].

#### Study design

**Fig. 1:**
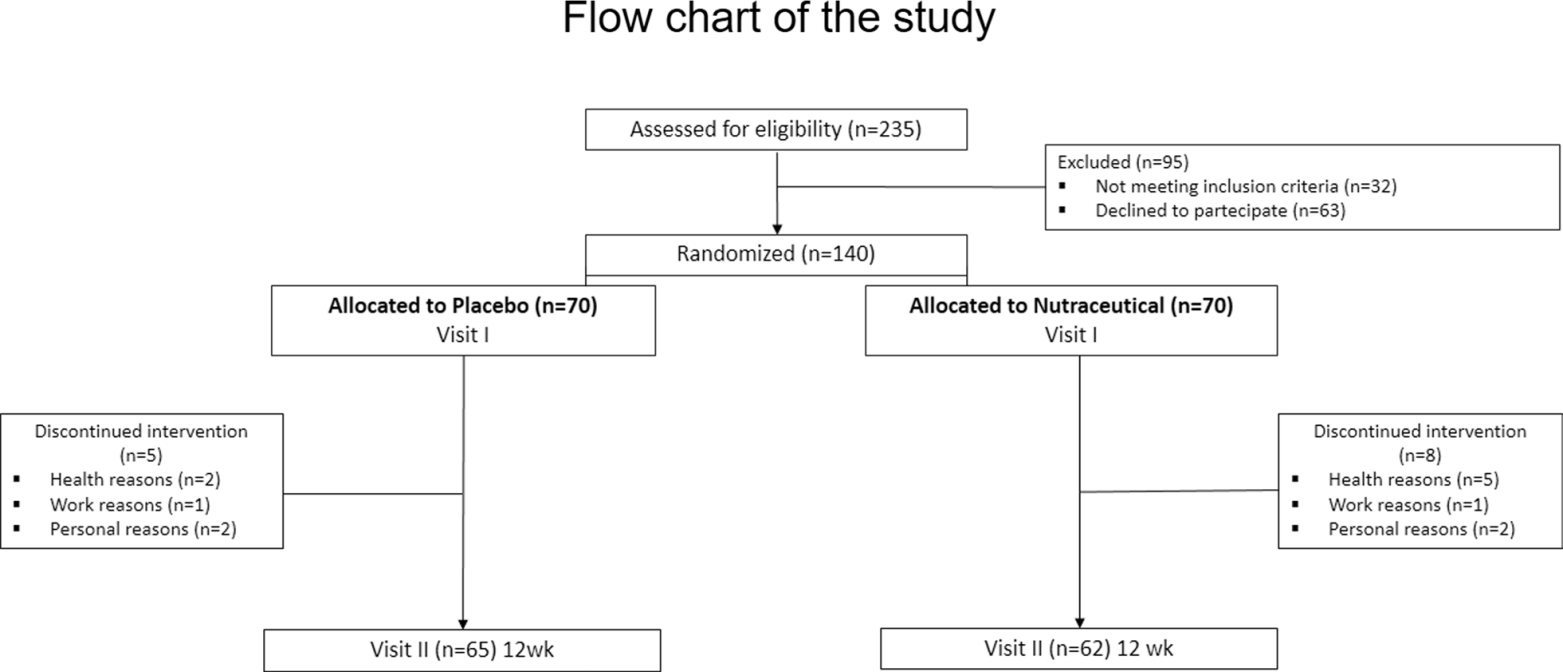
Flow-chart illustrating study population

Participants were randomly assigned in a 1:1 ratio to receive either Livogen Plus®, a nutraceutical containing curcumin complex, ω-3 PUFAs, BPF, artichoke leaf extract, black seed oil, pricoliv, GHS, SAMe and other natural bioactive components or a placebo for up to 12 weeks (Fig. 1; flow-chart of the study).

Participants are allocated with a simple randomization using computer-generated random numbers to the active or the control group. The active group receives six softgel capsules daily (3 for lunch and 3 for dinner) containing a combination of natural bioactive components for 12 weeks. The control group receives six softgel capsules daily (3 for lunch and 3 for dinner) of the placebo (dummy capsule) for 12 weeks.

The main outcome was a change in the amount of liver fat, assessed as “controlled attenuation parameter” (CAP) by VCTE, after 12 weeks of intervention between groups.

A registered dietician assigned participants to treatments. Both the participants and experimenters were blind to who received the nutraceutical or the placebo.

All participants signed written informed consent. The investigation conforms to the ethical principles outlined in the Declaration of Helsinki.

#### Livogen Plus® and placebo content

The nutraceutical tested was Livogen Plus® provided by Tishcon Corporation, NY, USA. We reported the specification sheet with all active ingredients of nutraceutical in the Table 1. Daily dose (6 softgel capsules) consisted of 667 mg of *Curcuma longa* extract complexed with γ-cyclodextrin (Cavacurmin®), 667 mg of refined fish oil concentrate (AlaskOmega®) that containing 213 mg of eicosapentaenoic acid (EPA) and 140 mg docosahexaenoic acid (DHA), 400 mg BPF and wild type *Cynara Cardunculus* extract (Bergacyn®) (Table 1). The nutraceutical softgel capsule also contained 334 mg of black seed oil of *Nigella Sativa* (ThymoQuin™), 267 mg of standardised fraction of root of *Picrorhiza kurroa* (Picroliv®), 200 mg reduced GHS (Opitac™) and SAMe, and other natural ingredients (Table 1). Livogen Plus® was made using components that are considered Generally Recognized As Safe (GRAS) (https://www.fda.gov/food/food-ingredients-packaging/generally-recognized-safe-gras). The placebo softgel capsules contained maltodextrins.

**Table 1:** Chemical composition of nutraceutical capsules

Ingredients	Amount per serving (mg)
Cavacurmin® (turmeric Ext. complexed with γ-cyclodextrin)	667
AlaskOmega® (refined fish oil concentrate)	667
EPA (eicosapentaenoic acid) TG	213
DHA (docosahexaenoic acid) TG	140
Bergacyn®	400
Black Seed Oil-ThymoQuin™ (*Nigella sativa*) 3% thymoquinone	334
Picroliv® (*Picrorhiza kurroa*) root	267
Opitac™ (reduced glutathione)	200
SAMe (as *S*-adenosyl-l-methionine)	200
Artichoke leaf Ext. (*Cynara scolymus* Sicc) 15% caffeoylquinic acids	167
Indole-3-carbinol	167
Silybin phospholipids (40% silybin)	167
Milk thistle fruit dry Ext. (80% silymarin)	127
Tulsi leaf Ext. aka Holy Basil (*Ocimum tenuiflorum* Lorum) 2.5% ursolic acid	50
Luteolin (*Sophora japonica* flower Ext.)	50
Schisandra berry (PE) (2% schizandrin)	33
Mixed tocotrienols complex (EVNol™)	33
Livinol® (*Garcinia indica* extract)	33
Dandelion root PE (4:1) (*Taraxacum*-officinale)	33
Natural astaxanthin (Astazine®)	8

The tested products had a similar macronutrient composition (i.e. per softgel, Total energy: ~ 7 kcal; Carbohydrates: 0.36 g; Proteins: 0.20 g; Lipids: 0.40 g). The nutraceutical and placebo softgels were similar in shape, size, and color. All capsules were put into bottles containing 180 capsules (for 1 month). All participants received three bottles that corresponded to approximately 13 weeks of intervention. Patient interviews and counting the number of capsules returned at the end of the study visit were used to estimate the treatment adherence.

#### Dietary intake

We assessed dietary intake with a food frequency questionnaire (FFQ) [15] at the baseline and after 12 weeks. In this RCT, each patient received written and oral advice to adhere to a Mediterranean Diet by a registered dietician [44]. The body weight loss was not an objective of the study. We thus prescribed the dietary recommendation to promote health-centered behaviors and not for study aims. Energy restriction was not advised for any of the treatment groups. The macronutrient profiles was about 50–55% range for carbohydrate, 20–30% for fats and 15–20% for protein, with a protein recommendation of 1 g/kg of ideal body weight [44].

The registered dietician suggested that a variety of aliments from the four food categories (grain products; vegetables and fruits; fish/meat; milk and derivatives) should be consumed, as well as more fresh unprocessed foods and legumes, extra virgin olive oil as the preferred dietary fat and fewer refined products. Thus, all participants received a printed copy of the dietary recommendation. Additional file 1: Table S1 shows the servings of various food groups recommended for this RCT.

#### Anthropometric measurements and cardiovascular risk factors assessment

Body weight was assessed on a calibrated digital scale (Tanita BC-418MA model) accurate to 0.1 kg, and standing height was measured with a stadiometer (seca 213 model) accurate to 0.1 cm. Anthropometric measurements (waist and hip circumferences) were also assessed, and body mass index (BMI) was calculated. We defined obesity by a BMI ≥ 30 kg/m^2^, and android obesity as a waist-hip-ratio (WHR) above 0.90 for males and above 0.85 for women [45].

The distinct cardiovascular (CV) risk factors was evaluated from patient interview and clinical record [15, 44, 46]. Systemic blood pressure (BP) was measured at the beginning and at the end of the study [15, 44]. The following criteria were used to define and exclude diabetes: antidiabetic drugs or fasting glycaemia ≥ 126 mg/dL [15, 44]. We defined Metabolic Syndrome (MS) according to National Cholesterol Education Program’s (NCEP) Adult Treatment Panel III report (ATP III) [47]. We identified participants with at least three or more abnormalities as having MS [47].

#### Vibration-controlled transient elastography for NAFLD diagnosis

Measurements of CAP and liver stiffness (LS) by VCTE (Fibroscan®; Echosense SASU, Paris, France) are non-invasive assessments for the diagnosing and staging of NAFLD and liver fibrosis [15, 48]. Both CAP and LS scores were measured in the same liver parenchyma volume at the same time. All subjects were examined using the 3.5 MHz standard M probe on the liver right lobe through intercostal spaces with the subject laying supine and the right arm behind the head to enable access to the abdomen right upper quadrant. The probe transducer’s tip was positioned on the skin between the ribs, at the level of the liver’s right lobe.

LS was expressed by the median value (in kPa) of ten measurements performed between 25 and 65 mm depth. For the study, we included only results with 10 valid shots and interquartile range (IQR)/median LS ratio < 30%. The cut-off value for the diagnosis of liver fibrosis was LS > 7 kPa.

The CAP algorithm is unique to this device, thus we only used the M probe to determine the CAP score. Each patient had 10 successful measurements, and only subjects with ten successful acquisitions were considered for this study.

The success rate was obtained by dividing the total number of successful measurements by the total number of measurements. The ratio of the IQR of LS to the median (IQR/M LS) was measured as a variability indicator. The CAP score was the median of single measurements with a value between 100 and 400 decibels per meter (dBm–). An indicator used to determine the variability of the final CAP was the ratio between IQR and the median CAP value (IQR/M CAP).

The same operator performed all scans. The diagnosis of NAFLD was based on a CAP score ≥ 247 dB/m. In addition, we categorized patients into 3 steatosis severity levels: CAP score between 247 and 268 dB/m for the diagnosis of S1 grade (mild), CAP between 269 and 280 dB/m for the diagnosis of S2 grade (moderate), and CAP ≥ 296 dB/m for the diagnosis of S3 grade (severe) [15, 48].

#### Biochemical evaluation

Venous blood was collected after fasting overnight into vacutainer tubes (Becton & Dickinson, Plymouth, England) and centrifuged within 4 h. Serum glucose, insulin, triglycerides (TG), total cholesterol (TC), high density lipoprotein cholesterol (HDL-C), albumin, ALT, AST, γGT, c-reactive protein (CRP) and creatinine were measured using a chemiluminescent immunoassay according to the manufacturer’s instructions on a COBAS 8000 (Roche, Switzerland), at baseline and after 12 weeks, according to the manufacturer’s instructions. Low-density lipoprotein cholesterol (LDL-C) values were calculated by following Friedewald formula: LDL-C = TC − [HDL-C + (TG/5)] [49].

We considered the following cut-off value at baseline for the definition of “low HDL-C” in men HDL-C < 40 mg/dL and in women HDL-C < 50 mg/dL [47].

We calculated the homeostatic model assessment (HOMA) index to assess the β-cell function and IR from serum fasting glucose and insulin levels [50].

The serum levels of tumor necrosis factor α (TNF-α), interleukin-1β (IL-1β), and interleukin-6 (IL-6), were measured by ELISA Kit (Life Technologies Italia, Monza, Italia) according to the instructions of the manufacturer.

The biological antioxidant potential (BAP) level was measured as a marker of oxidative stress. Measurement of the BAP concentrations (Diacron International, Grosseto, Italy) was performed using a spectrophotometer, as reported previously [51].

#### Safety parameters and adverse events

We assessed different parameters of global health, such as systemic BP as well as serum creatinine, glucose, lipids, and transaminases.

A patient-reported outcome questionnaire was used to report the adverse events (AEs). The questionnaire was performed to review any new symptoms after entry into the study that could be related to the treatment. The type and severity of the AEs were also evaluated.

### In vitro study

#### Polyphenols extraction and antioxidant activity

We also investigated the direct effect of nutraceutical, compared to placebo, in a cellular model of hepatic steatosis. Polyphenols from nutraceutical and placebo capsule were extracts. At one capsule of nutraceutical and placebo was added with 50 mL of water:ethanol mixture (1:3). Then, the extracts were first purified by centrifuge at 2500 rpm/5 min, followed by two centrifuges of 20×*g*/8 min. Subsequently the extracts were characterized for the antioxidant activity. The antioxidant activity of nutraceutical and placebo extract was quantified by DPPH assay, to investigate the capability of two extracts to inhibit free radicals. DPPH solution and l-ascorbic acid solution (5 mg/mL) were used as negative and positive controls, respectively (x) [52]. All quantifications were performed with a spectrophotometer (UV–Vis Genesys 150®-ThermoScientific).

#### Cell culture and treatments

Rat hepatoma cells, McA R7777 were obtained from American Type Culture Collection ATCC. The cells were maintained in DMEM (Sigma Aldrich, ST. Louis, USA), supplemented with 10% FBS with 1% penicillin streptomycin (PAA, Linz, Austria) and 1% sodium pyruvate (PAA, Linz, Austria), at 37 °C in 5% CO2, harvested by trypsinization, and subcultured twice weekly. McA Rh-7777 hepatic cells have been treated with 50 µM of oleic acid (OA) (Sigma Aldrich, St. Louis, USA) conjugated to fatty acid-free bovine serum albumin (BSA) and 25 and 12.5 µg/mL of nutraceutical and placebo extract for 24 h. We evaluated cell viability on McA RH7777 cells that were seeded at a density of 10,000 cells/well in 96-well plates. Cell viability was determined by 3-(4,5-dimethylthiazol-2-yl)-2,5-diphenyltetrazolium bromide (MTT) assay. Briefly, MTT (Sigma, St. Louis, MO, USA) solution (5 mg/mL) was added to each well and incubated at 37 °C for 4 h. The supernatant was then removed and replaced by 100 mL of DMSO. The optical density (OD) was measured at a wavelength of 570 nm.

#### Oil Red O (ORO) staining

For evaluate intracellular lipid content, McA Rh-7777 cells were seeded in a coverslip at a density of 5 × 10^4^ cells/well in 24-well plates. After treatments the cells were washed with PBS, and fixed with 2% paraformaldehyde for 5 min. Intracellular lipids were stained with Oil Red O solution (sigma, St. Louis; MO, USA) for 20 min. Cell nuclei were stained with Mayer reagent for 5 min. All the staining procedure was carried out at room temperature by protecting the samples from the direct light. Images were acquired with microscope (Leica DM 1000 LED) with a digital camera (LEICA ICC50 W) at 20× magnification. We quantified the images using ImageJ software (v.1.52 h, NIH).

### Statistical analysis

Data are reported as mean ± standard deviation (SD).

It was considered a mean CAP value of 268.6 ± 52 dB/m for adults with NAFLD [53]. Thus, to detect a CAP score reduction of at least 12%, with an effect size (ES = mean CAP difference/baseline SD) of 0.62, with 80% power on a two-sided level of significance of 0.05, a minimum of 44 subjects for each group were required. Considering a 35% of drop-out [54], we enrolled 140 patients (Fig. 1).

We tested continuous data for normal distribution with the Kolmogorov–Smirnov or Shapiro–Wilk tests. A Chi-square test was performed to analyse the prevalence between groups and an independent unpaired samples t test was used to compare the difference between means. Specifically, we calculated the changes in variables and compared the means of these changes between intervention groups. Changes in the clinical characteristics from baseline to follow-up (within group variation) were analysed using paired Student’s t test (two tailed). We used the Fisher’s Least Significant Differences (*LSD*) adjustment method for multiple testing correction (General Linear Model-GLM).

Nonparametric tests (Mann–Whitney U test and Wilcoxon signed-rank test) were used to evaluate the differences in BAP, IL-1β, IL-6 and TNF-α value between and within groups. Both intention-to-treat (ITT) and per-protocol (PP) analyses were performed. PP analysis was performed only on participants taking 80% or more of the prescribed treatment.

To better describe the response to nutraceutical, we performed some post-hoc analyses (ITT; PP; men; women; over 60 years; with age ≤ 60 years; with baseline low HDL-C; with baseline IR, and with AST reduction at follow-up) where the CAP score change was analyzed. For the post-hoc analyses, we performed an independent unpaired samples t test to compare the difference in CAP score change (both absolute value and %) between groups. Furthermore, we used a Chi-square test to compare the improvement in the liver steatosis grade between groups. The GLM was used to adjust both the CAP score reduction and liver steatosis grade improvement for potential confounders.

A p-value was significant if < 0.05 (two-tailed). All analyses were performed with SPSS 22.0 for Windows (IBM Corporation, New York, NY, United States).

For the in vitro study, data resulted from a mean of at least two independent experiments and were analyzed with GraphPad Prism 5.0 software using a two-tailed Student’s t test.

## Results

One hundred and twenty-seven participants completed the study. Five subjects stopped treatment in the placebo group and eight subjects in the nutraceutical group (Fig. 1). One person was lost within 12 weeks due to allergy, one to diarrhoea, and three to abdominal discomfort (bloating, pain or cramps) in the active group. In the placebo group, one individual was lost due to diarrhoea, and one to abdominal discomfort. In addition, in both groups, two individuals discontinued the RCT for personal reasons, and one for work reasons (Fig. 1).

The mean age of the population was 54 ± 9 years. A total of 74 (53%) were male. The mean basal BMI and CAP score was 28.9 ± 4 kg/m^2^ and 297 ± 32 dB/m, respectively.

### Baseline demographic and clinical characteristics of participants according to the treatments

Table 2 shows the basal anthropometric and clinical characteristics of participants according to the allocation (n = 140). The groups were comparable for all the characteristics except for serum TC levels that were statistically higher in the active group than in the control group (TC: 204 ± 31 vs. 191 ± 33 mg/dL, p = 0.018; respectively, n = 70 each group). Approximately 60% of the participants had a severe hepatic steatosis (S3 grade).

**Table 2:** Baseline demographic and clinical characteristics of participants according to the treatments

Variables	Placebo (n = 70)	Nutraceutical (n = 70)	*p*-value
Age (years)	54 ± 8	54 ± 10	0.96
Weight (kg)	79 ± 11	77 ± 12	0.31
BMI (kg/m^2^)	29.3 ± 3	28.7 ± 4	0.33
WHR	0.97 ± 0.07	0.96 ± 0.07	0.46
FM (kg)	25 ± 7	24 ± 7	0.73
SBP (mmHg)	118 ± 12	117 ± 13	0.62
DBP (mmHg)	77 ± 10	76 ± 9	0.81
CAP score (dB/m)	300 ± 32	294 ± 32	0.28
IQR	10 ± 5	11 ± 5	0.32
Stiffness (kPa)	4.6 ± 1.2	4.7 ± 1.0	0.47
IQR	15 ± 6	16 ± 7	0.65
Glucose (mg/dL)	89 ± 9	91 ± 10	0.11
Insulin (mU/L)	13 ± 10	12 ± 7	0.49
HOMA-IR	2.95 ± 2.2	2.82 ± 1.7	0.69
TC (mg/dL)	191 ± 33	204 ± 31	0.018
TG (mg/dL)	118 ± 51	137 ± 83	0.10
HDL-C (mg/dL)	50 ± 11	52 ± 12	0.30
Albumin (g/dL)	4.4 ± 0.4	4.4 ± 0.5	0.95
AST (IU/L)	22 ± 11	21 ± 8	0.46
ALT (IU/L)	26 ± 23	23 ± 15	0.43
γGT (UI/L)	28 ± 22	23 ± 14	0.10
Creatinine (mg/dL)	0.85 ± 0.1	0.82 ± 0.2	0.28
CRP (mg/L)	3.7 ± 2	4.2 ± 4	0.27
Prevalence			
Gender (male, %)	56	50	0.61
Menopause (%)	77	66	0.41
Physical activity (%)	46	54	0.39
Smokers (%)	39	31	0.47
Obesity (%)	37	37	1
Metabolic syndrome (%)	16	17	1
Android obesity (%)	91	97	0.27
Insulin resistance (%)	46	45	1
Hypertension (%)	40	37	0.86
Hyperlipidemia (%)	47	50	0.86
Antihypertensive drugs (%)	36	31	0.72
Antiplatelet agents (%)	11	9	0.77
Lipid-lowering agents (%)	21	14	0.37
Liver steatosis S1 grade (%)	14	24	0.39
Liver steatosis S2 grade (%)	24	16	
Liver steatosis S3 grade (%)	61	60	
Liver fibrosis (%)	0	0	/

Difference between means by unpaired samples t test

*BMI* body mass index, *WHR* waist to hip ratio, *FM* fat mass, *SBP* systolic blood pressure, *DBP* diastolic blood pressure, *CAP* controlled attenuation parameter, *IQR* interquartile range, *HOMA-IR* homeostatic model assessment of insulin resistance, *TC* total cholesterol, *TG* triglycerides, *HDL-C* high density lipoprotein cholesterol, *AST* aspartate aminotransferase, *ALT* alanine aminotransferase, *γGT* gamma glutamyltransferase, *CRP* C-reactive protein

### Dietary intake assessment

The Additional file 2: Table S2 shows the dietary intake assessment of the participants according to the allocation. At baseline, the nutrient profile of the two groups was comparable (Additional file 2: Table S2). Additional file 3: Table S3 shows nutrient profile assessment at baseline and dietary intake changes during the treatment period of participants who completed the study (12 weeks). At the baseline of treatment, the two groups were comparable. At the end of the intervention, we found a statistically significant reduction in the intake of animal proteins and dietary cholesterol, and an increase in the intake of vegetable proteins in the participants taking nutraceutical compared to placebo (Additional file 3: Table S3).

### Clinical characteristics changes at follow-up and outcome of the study

Table 3 shows the baseline and follow-up clinical characteristics of subjects who completed the study (12 weeks) according to the intervention group (n = 127). At baseline, the groups were comparable for all the characteristics except for serum TC and TG levels that were statistically higher in the nutraceutical group than in the control group (TC: 210 ± 39 vs. 191 ± 32 mg/dL, p = 0.015; TG: 133 ± 92 vs. 115 ± 49 mg/dL, p = 0.034; n = 62/65 respectively). CAP score values did not differ between the two groups, even after adjustment for baseline TG concentration (Table 3). DBP, insulin, and HOMA-IR, decreased only in the participants taking nutraceutical (HOMA-IR from 2.8 ± 1.7 to 2.4 ± 1.3 mg/dL, p = 0.023; Table 3). Serum HDL-C levels increased, and the CAP score, body weight, WHR, IL-1β and IL- 6 decreased in the participants taking nutraceutical as well as in subjects in the placebo group (in nutraceutical group, CAP score dropped from 294 ± 32 to 265 ± 40 dB/m, p < 0.001; Table 3). In addition, participants in the nutraceutical group at baseline had a lower BAP values than controls (p < 0.001; Table 3). At the end of the study, only these participants reported a significant increase in BAP levels (Table 3).

**Table 3:** Baseline and follow-up clinical characteristics of participants according to the treatments (intention to treat analysis)

Variables	Placebo (n = 65)			Nutraceutical (n = 62)			*p*-value (unpaired t test between basal values)
	Basal	Follow-up	*p*-value (paired t test)	Basal	Follow-up	*p*-value (paired t test)	
Weight (kg)	80 ± 11	79 ± 11	0.011	77 ± 12	76 ± 11	< 0.001	0.26
BMI (kg/m^2^)	29.5 ± 3	29.1 ± 4	0.006	28.4 ± 4	28.0 ± 4	< 0.001	0.11
WHR	0.96 ± 0.07	0.95 ± 0.07	< 0.001	0.96 ± 0.07	0.93 ± 0.06	< 0.001	0.62
FM (kg)	25 ± 7	25 ± 7	0.81	24 ± 7	24 ± 7	0.49	0.30
SBP (mmHg)	118 ± 12	118 ± 10	0.90	118 ± 13	117 ± 13	0.78	0.92
DBP (mmHg)	76 ± 9	76 ± 9	0.55	76 ± 9	74 ± 9	0.012	0.91
CAP score (dB/m)	302 ± 32	277 ± 42	< 0.001	294 ± 32	265 ± 40	< 0.001	0.16
*a*CAP score^a^ (dB/m)	303 ± 4	–	–	292 ± 4	–	–	0.055
Stiffness (kPa)	4.6 ± 1.2	4.7 ± 1.1	0.34	4.7 ± 1.0	4.6 ± 1.1	0.24	0.53
Glucose (mg/dL)	89 ± 9	90 ± 9	0.17	91 ± 10	91 ± 9	0.41	0.14
Insulin (mU/L)	13 ± 10	12 ± 7	0.26	12 ± 7	11 ± 5	0.023	0.44
HOMA-IR	3.0 ± 2.3	2.8 ± 1.7	0.36	2.8 ± 1.7	2.4 ± 1.3	0.023	0.64
TC (mg/dL)	191 ± 32	192 ± 31	0.49	204 ± 31	210 ± 39	0.13	0.015
TG (mg/dL)	115 ± 49	121 ± 57	0.31	142 ± 86	133 ± 92	0.29	0.034
HDL-C (mg/dL)	49 ± 11	52 ± 12	0.001	50 ± 11	52 ± 12	0.009	0.66
Albumin (g/dL)	4.4 ± 0.3	4.5 ± 0.4	0.20	4.4 ± 0.5	4.5 ± 0.4	0.27	0.50
AST (IU/L)	22 ± 12	20 ± 10	0.09	22 ± 8	21 ± 6	0.40	0.77
ALT (IU/L)	25 ± 24	24 ± 18	0.57	24 ± 15	24 ± 13	0.84	0.73
γGT (UI/L)	27 ± 20	24 ± 17	0.020	24 ± 15	25 ± 15	0.50	0.37
Creatinine (mg/dL)	0.85 ± 0.1	0.87 ± 0.1	0.046	0.83 ± 0.2	0.83 ± 0.2	0.74	0.45
CRP (mg/L)	3.7 ± 1.6	3.6 ± 1.2	0.55	4.2 ± 3.6	3.8 ± 1.9	0.36	0.34
BAP (μmol/L)	1998 ± 540	2169 ± 385	0.12	1590 ± 427	1707 ± 506	0.031	< 0.001
Cytokine evaluation							
IL-1β (pg/mL)	16.6 ± 5	14.7 ± 11	< 0.001	15.9 ± 3	13.8 ± 3	0.002	0.65
IL-6 (pg/mL)	9.2 ± 7	6.9 ± 1	< 0.001	8.1 ± 2	6.9 ± 1	< 0.001	0.75
TNF-α (pg/mL)	12.9 ± 9	13.1 ± 9	0.93	15.5 ± 24	12.1 ± 5	0.93	0.94

Difference between means by unpaired samples t test; within group variation by paired Student’s t test (two tailed); differences in BAP, IL-1β, IL-6 and TNF α by Mann–Whitney U test and Wilcoxon signed-rank test. Difference between means by unpaired samples t test with adjustment by General Linear Model

*BMI* body mass index, *WHR* waist to hip ratio, *FM* fat mass, *SBP* systolic blood pressure, *DBP* diastolic blood pressure, *CAP* controlled attenuation parameter, *HOMA-IR* homeostatic model assessment of insulin resistance, *TC* total cholesterol, *TG* triglycerides, *HDL-C* high density lipoprotein cholesterol, *AST* aspartate aminotransferase, *ALT* alanine aminotransferase, *γGT* gamma glutamyltransferase, *BAP* biological antioxidant potential, *IL-1β* interleukin-1β, *IL-6* interleukin-6, *TNF-α* tumor necrosis factor α

^a^*a*CAP score adjusted for serum triglycerides at baseline

**Fig. 2:**
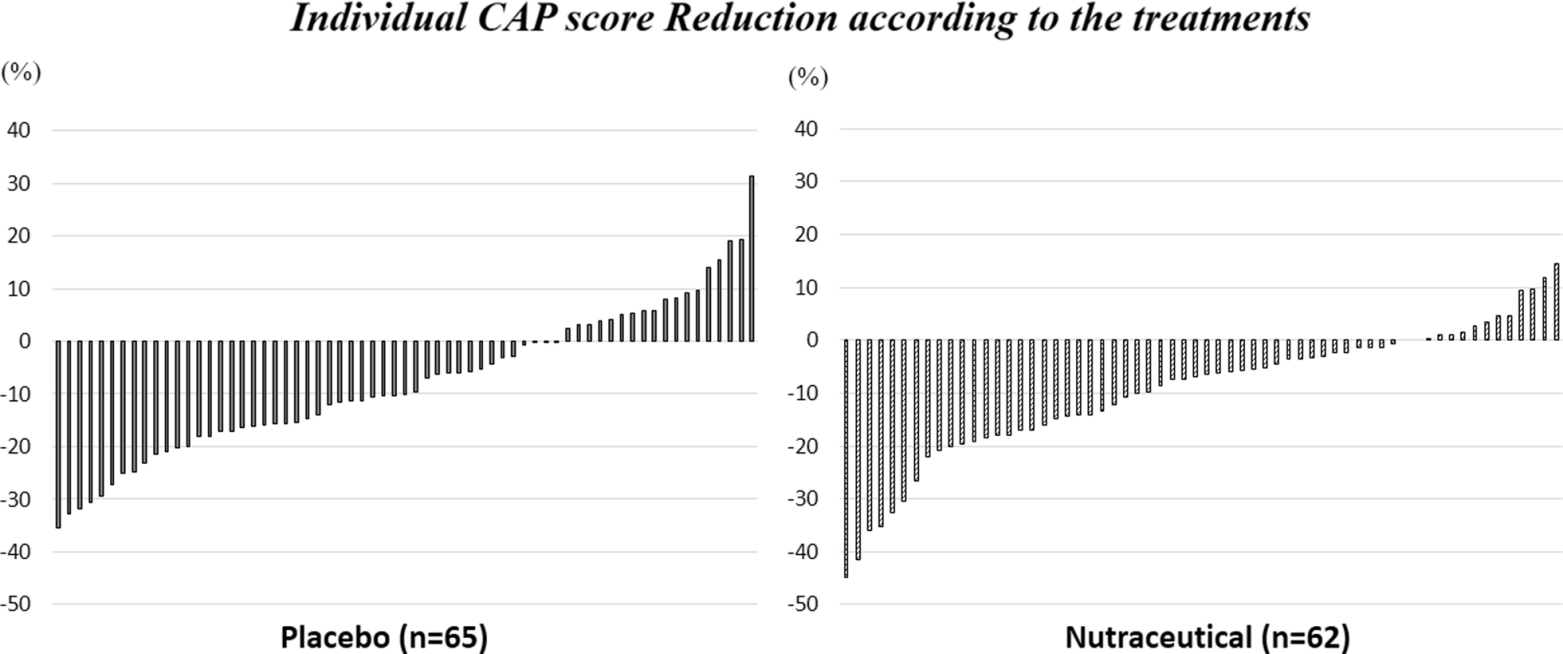
Individual CAP score reduction according to the treatments after 12 weeks

Figure 2 shows the individual CAP score change for the participants in each intervention group. The changes in the clinical parameters at the follow-up are shown in Additional file 4: Table S4. The change in the clinical parameters and in serum concentration of BAP, CRP, IL-1β, IL-6 and TNF-α did not differ between groups (Additional file 4: Table S4). In the placebo group, we found a significant reduction in γGT levels compared to the active group (Additional file 4: Table S4). The change in the CAP score did not differ between groups (Additional file 4: Table S4).

**Fig. 3:**
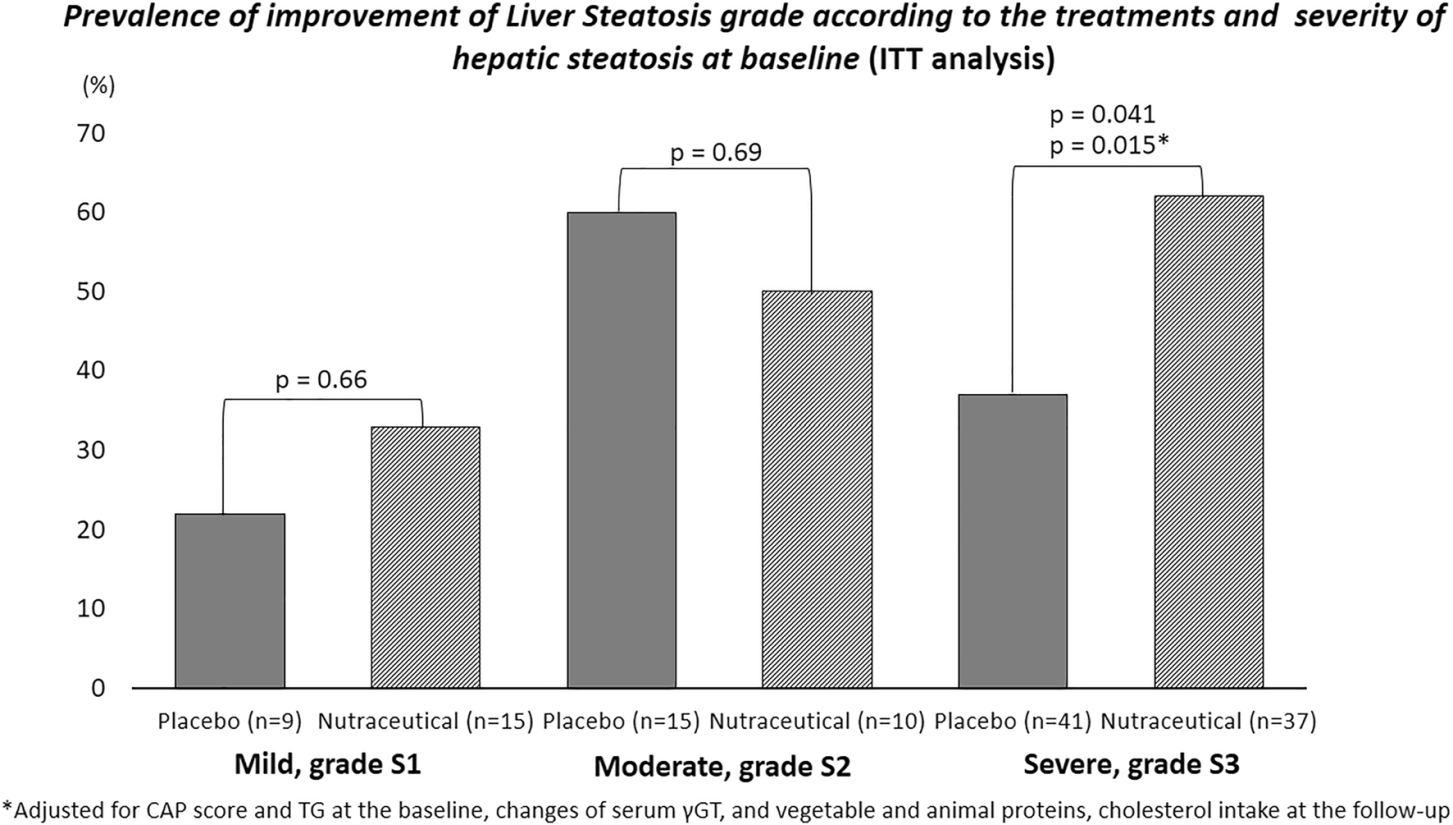
Prevalence of improvement of Liver Steatosis grade according to the treatments and severity of hepatic steatosis at baseline (ITT analysis). Prevalence between groups by Chi-square test with adjustment by General Linear Model

Figure 3 shows the prevalence of improvement of liver steatosis grade according to the treatments and severity of NAFLD at baseline. In the group of participants with severe hepatic steatosis (S3 grade), we found that subjects taking nutraceutical capsules had a significantly greater improvement in the stage of liver steatosis compared to placebo (62% vs. 37%, p = 0.041; respectively). The improvement of NAFLD grade remained statistically significant even after adjustment for confounding variables (p = 0.015; Fig. 3).

### Subgroup analysis

We performed several statistical analyses in the participants taking more than 80% of the prescribed treatment (PP analysis). Results of PP analysis are shown in Additional file 5: Table S5, Additional file 6: Table S6 and Additional file 7: Table S7. The prevalence of improvement of hepatic steatosis grade according to the treatments and severity of NAFLD at baseline is shown in Additional file 8: Fig. S1 (PP analysis).

Finally, the changes in the clinical parameters in subgroups according to the interventions are shown in Table 4. In the ITT analysis, we found that the nutraceutical supplementation significantly lowered CAP score compared to the placebo after adjustment for CAP score and TG at the baseline, changes of serum γGT, vegetable and animal proteins, cholesterol intake at the follow-up (CAP score: − 34 ± 5 vs. − 20 ± 5 dB/m, p = 0.045; respectively) (Table 4).

**Table 4:** Changes in clinical parameters in the subgroups according to the treatments

ITT			
Variables	Placebo (n = 65)	Nutraceutical (n = 62)	*p*-value
CAP score (dB/m)^a^	− 20 ± 5	− 34 ± 5	0.045
CAP (%)^a^	− 6.4 ± 2	− 11.2 ± 2	0.049
Improvement (%)^a^	40	53	0.09

PP			
Variables	Placebo (n = 54)	Nutraceutical (n = 55)	*p*-value
CAP score (dB/m)^b^	− 18 ± 5	− 37 ± 5	0.018
CAP (%)^b^	− 5.9 ± 2	− 11.7 ± 2	0.034
Improvement (%)^b^	39	55	0.041

Men			
Variables	Placebo (n = 29)	Nutraceutical (n = 30)	*p*-value
CAP score (dB/m)^b^	− 17 ± 7	− 37 ± 7	0.07
CAP (%)^b^	− 4.6 ± 2	− 12.1 ± 2	0.042
Improvement (%)^b^	31	50	0.019

Age ≤ 60 years			
Variables	Placebo (n = 41)	Nutraceutical (n = 36)	*p*-value
CAP score (dB/m)^b^	− 18 ± 6	− 36 ± 7	0.05
CAP (%)^b^	− 5.6 ± 2	− 12.3 ± 2	0.044
Improvement (%)^b^	44	56	0.12

Insulin resistance (HOMA IR > 2)			
Variables	Placebo (n = 36)	Nutraceutical (n = 32)	*p*-value
CAP score (dB/m)^b^	− 20 ± 6	− 31 ± 6	0.21
CAP (%)^b^	− 5.9 ± 2	− 10.1 ± 2	0.14
Improvement (%)^b^	31	56	0.031

Low HDL-C			
Variables	Placebo (n = 17)	Nutraceutical (n = 18)	*p*-value
CAP score (dB/m)^b^	− 6 ± 10	− 38 ± 10	0.05
CAP (%)^b^	− 1.1 ± 3	− 12.5 ± 3	0.038
Improvement (%)^b^	24	61	0.05

AST reduction			
Variables	Placebo (n = 30)	Nutraceutical (n = 23)	*p*-value
CAP score (dB/m)^b^	− 13 ± 8	− 47 ± 9	0.007
CAP (%)^b^	− 3.7 ± 3	− 15.1 ± 3	0.006
Improvement (%)^b^	33	58	0.043

Difference between means by unpaired samples t test with adjustment by General Linear Model

*CAP* controlled attenuation parameter

^a^Adjusted for CAP score and TG at the baseline, changes of serum γGT, and vegetable and animal proteins, cholesterol intake at the follow-up

^b^Adjusted for TG at the baseline, changes of serum γGT, and vegetable proteins intake at the follow-up

After adjustment, the CAP score reduction (%) was similar in the ITT analysis and in PP analysis (− 11.2% vs. − 11.7% in ITT vs. PP analysis).

In PP analysis, CAP score reduction was higher in participants aged 60 or less and in men rather than ITT analysis (Table 4). However, after adjustment, the CAP score reduction was even greater in subjects with low HDL-C at baseline (nutraceutical: − 12.5 ± 3% vs. placebo: − 1.1 ± 3%, p = 0.03) and in those with AST reduction at follow-up (nutraceutical: − 15.1 ± 3% vs. placebo: − 3.7 ± 3%, p = 0.006) (Table 4). In addition, we found a greater prevalence of improvement of NAFLD degree after taking the nutraceutical in the PP analysis, in men, and in the participants with low basal HDL-C, or with IR or with AST reduction (Table 4, Additional file 9: Fig. S2).

### Adverse events

**Fig. 4:**
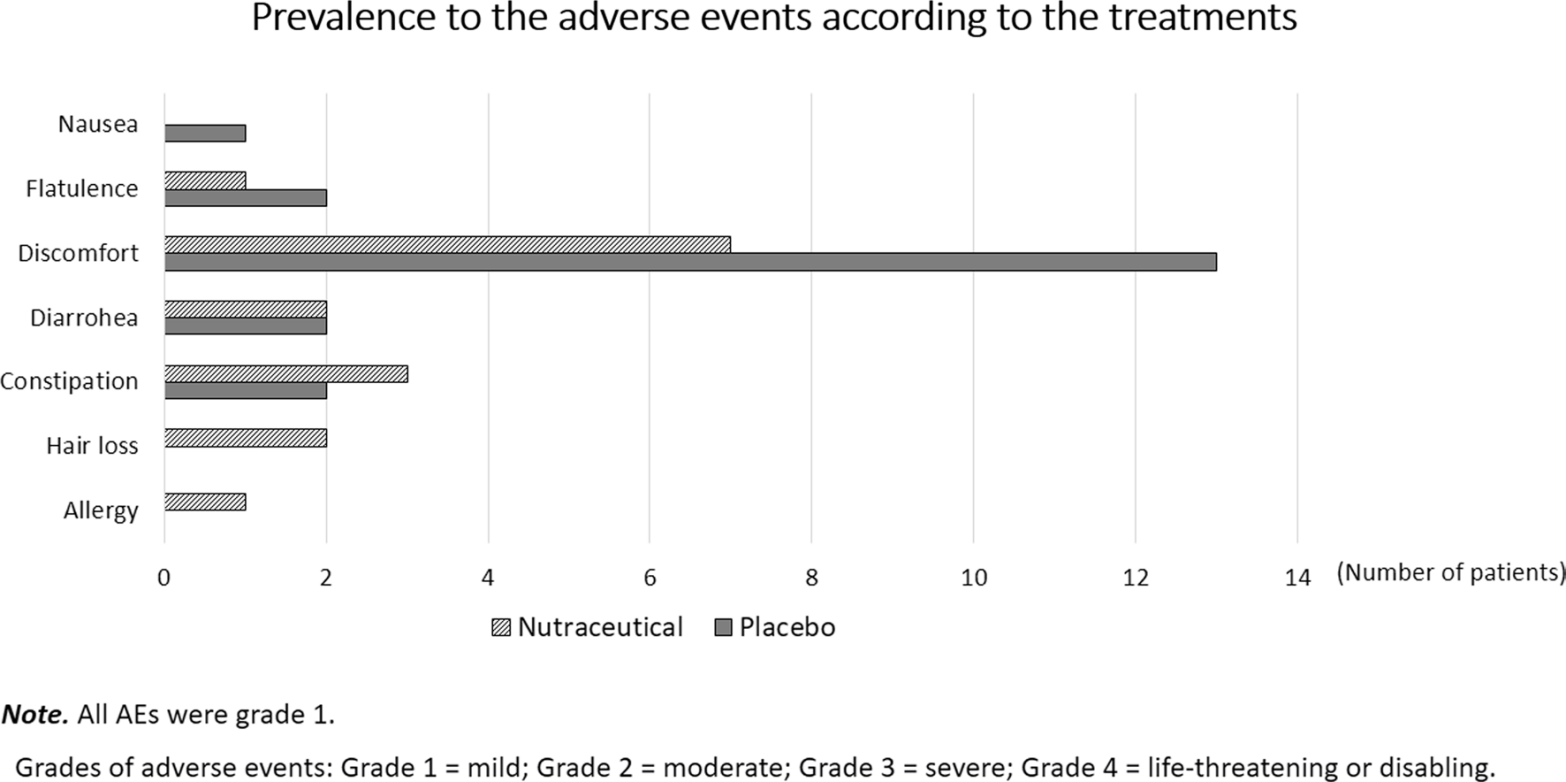
Prevalence to the adverse events according to the treatments after 12 weeks

The nutraceutical softgel capsules were well tolerated and did not cause any change in systemic blood pressure, creatinine, glucose, lipids and liver enzymes (AST and ALT) values (Additional file 5: Table S5). The AEs were all of grade 1 (mild), and we reported them in the Fig. 4.

### Livogen extract reduced intracellular neutral lipid in McR-Rh777

McA-Rh7777 cells were incubated with 50 µM of oleic acid and an increasing dose of nutraceutical or placebo (12.5 and 25 µg/mL) for 24 h. Given that nutraceutical or placebo was dissolved directly in the media, cell media was used as a negative control. Cell viability was measured by MTT assay. This assay showed that both Livogen Plus® and placebo did not affect cell viability at 24 h as compared to the control (Additional file 10: Fig. S3).

**Fig. 5:**
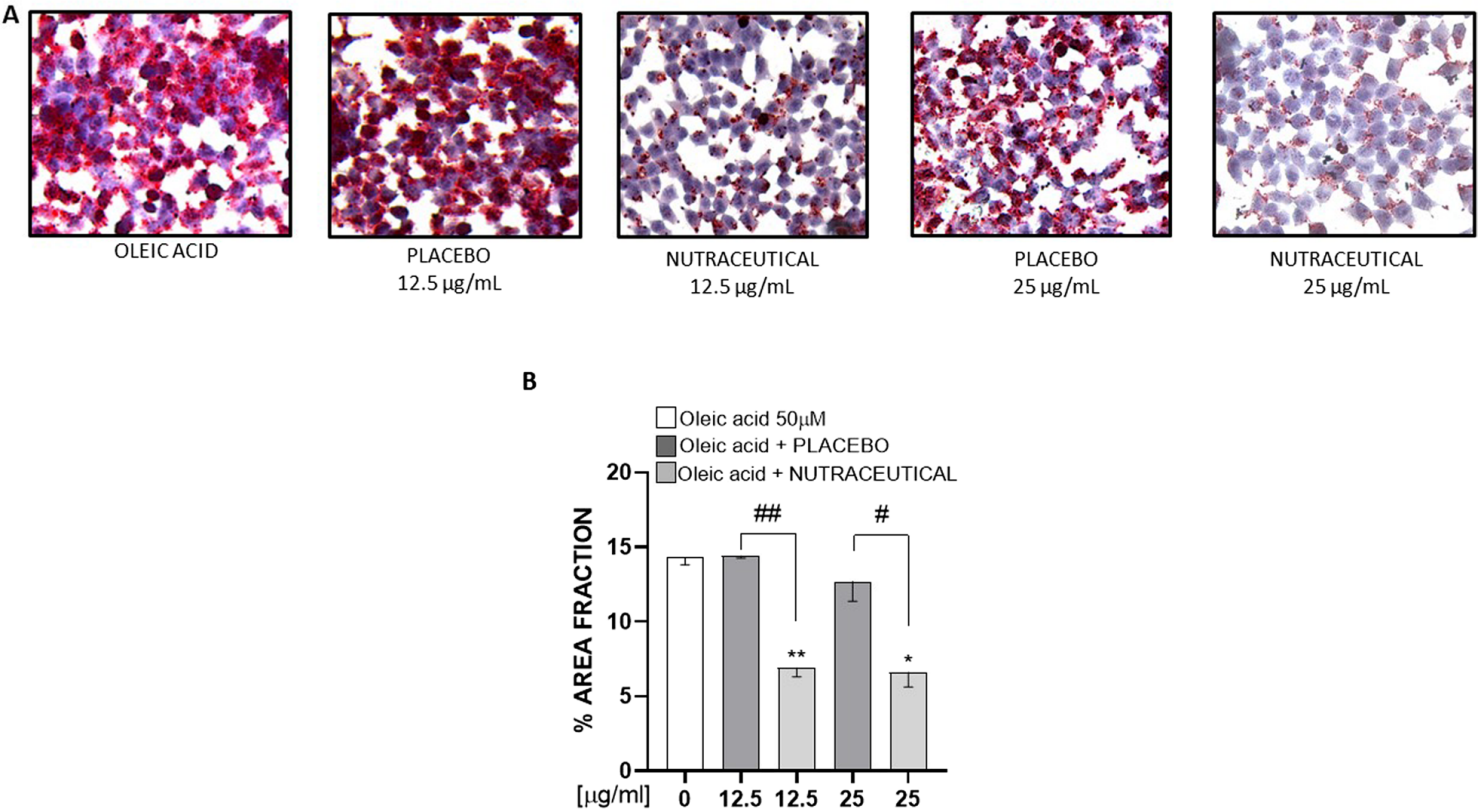
Nutraceutical polyphenols extract decreases intracellular neutral lipid in McR-Rh7777. Rat hepatoma cell line McARh-7777 was cultured in 2D and incubated with 50 µM of oleic acid and different concentrations of nutraceutical (25–12.5 µg/mL) or placebo in regular medium without FBS for 24 h. Intracellular lipid content was measured by Oil red-O staining, and representative images were acquired at 40× magnification (**A**). ORO area quantified by Image J (**B**) and showed a significant decreased of intrahepatic lipids content. Data shown as mean ± SD in all groups. Statistical analysis: *p < 0.05; **p < 0.01 (Student t Test) vs 0; ^#^< 0.05 ^##^p < 0.01 (Student t test) vs. placebo

To evaluate the neutral intracellular lipid content in hepatocytes, we incubated McA-Rh7777 cells with increasing concentration of nutraceutical and placebo extract by using Oil Red O staining. Cells were incubated with 25 µg/mL or 12.5 µg/mL of nutraceutical and placebo extract for 24 h and intracellular lipid content was then examined. The nutraceutical extract significantly decreased the intracellular lipid content both in comparison with oleic acid (Student t’ test: p = 0.0103 and p = 0.0057, respectively) and in comparison with Placebo extract (Student t’ test: p = 0.0352 and 0.0035, respectively) (Fig. 5).

Furthermore, it was found that the antioxidant activity of nutraceutical extract has a significant inhibitory power of free radicals compared to placebo extract (82.9% vs. − 3.6%, p < 0.001; respectively) (Additional file 11: Table S8).

## Discussion

The efficacy and safety of the present nutraceutical compound could be explained by the combination of its components: curcumin, ω-3 PUFAs, BPF, artichoke leaf extract, black seed oil, pricoliv, GHS, SAMe and other natural ingredients. Starting from the knowledge of the beneficial effects of each abovementioned compound on liver fat content, we hypothesized that a new dietary supplement comprising of a mixture of molecules extracted from bergamot, fish, vegetables and plants could reduce liver steatosis by its metabolic, antioxidant and anti-inflammatory properties and also through their synergistic effect.

Among the nutraceuticals currently available, the present product (namely Livogen Plus®) contains the largest number of antioxidants species.

For the first time, this new nutraceuticals combination significantly reduced the CAP score by 11.2% (CAP score absolute: − 20 ± 5 and − 34 ± 5 dB/m in the placebo and active treatment, respectively) after 12 weeks (Table 4). Subgroup analysis revealed that the highest reduction in CAP score (by ~ 12%) could be found in the participants with low HDL-C at baseline (Table 4). Most important, we found that in subjects with the greatest reduction in AST, the greatest reduction in CAP was achieved (Table 4). This finding is in line with the concept that AST is an enzyme that is found mainly in the liver while ALT is found in the liver, brain, pancreas, heart, kidneys, lungs, and skeletal muscles. As a screening test for liver disease, ALT has a very low specificity.

The reduction observed in the CAP score was in the range of those obtained in one RCT with a combination product containing *Cynara cardunculus* and *Citrus bergamia* extracts in a population similar to ours (i.e. non-diabetic adults with NAFLD) [15]. In that study, active treatment compared with placebo was associated with a significant reduction in CAP score (~ − 27 vs. ~ − 48 dB/m in the placebo and nutraceutical group, respectively) [15]. However, in that study there was a lower prevalence of severe steatosis grade (S3) than in our study (~ 44% vs. ~ 60%, respectively) [15]. It is very interesting to note that our data also confirms the evidence that individuals with baseline severe hepatic steatosis have the greatest benefit from nutraceutical treatment. Indeed, 62% of participants with severe NAFLD in the active group had an improvement in the degree of liver steatosis compared to 37% of individuals in the placebo group (Fig. 3).

The efficacy of this nutraceutical on NAFLD is also corroborated by the in vitro study. Indeed, the polyphenols extract from nutraceutical significantly reduced the intracellular lipid content compared to the control groups in hepatocytes (Fig. 5). In addition, as expected, the nutraceutical extract had antioxidant properties (Additional file 11: Table S8).

Furthermore, our results are confirmed by several previous pre-clinical and clinical studies of NAFLD with the molecules contained in the nutraceutical.

Nutraceuticals used in the Livogen formula are generally considered safe and at low risk of adverse effects. Many ingredients in the Livogen Plus® softgel capsules are provided at dosages that can be supported by the available evidence. In order to reach these dosages, the active group receives six softgel capsules daily (3 for lunch and 3 for dinner, as well as placebo) for 12 weeks. Furthermore, each component of Livogen Plus® is safe and well tolerated among humans (Fig. 4, see Additional file 12: Table S9 “Safety assessment of Livogen”). The abdominal discomfort was reported by thirteen participants in the control group and only seven subjects in the nutraceutical group, all of grade 1 (mild). The specific ingredients used, including the amount of active component within each ingredient, in combination with one another, have a major influence on the product’s overall effectiveness. Research verified dosages of vitamin E often range from 400 to 1000 IU per day or higher [55]. The UL of vitamin E is 1000 mg for adult men and women. This means that, at the dosages of Livogen in the enrolled individuals, vitamin E remain at a dose considered safe by research studies.

In our study, EPA and DHA are at a safe dosage of approximately 700 mg near to the amount used in other research studies [55, 56]. Bergacyn® showed significant safety and efficacy in a clinical trial at 600 mg/day, while in our study we used 400 mg/day of Bergacyn® [16].

This means that, in our study, the dosages of each component of Livogen Plus® remains close to the dose of previous research studies, in term of safety and efficacy. In a similar way the dosages of other nutrients of Livogen were chosen [19, 57–61] (Additional file 12: Table S9).

Pre-clinical and clinical studies showed that curcumin extracted from *Curcuma longa* reduces body weight, transaminases, lipids, [14, 62–64] and was efficacious in patients with a high severity grade of hepatic steatosis [63, 65].

The synergistic effect of *Cynara cardunculus* and *Citrus bergamia* extracts has also been shown to be effective in lowering liver fat content, body weight, transaminases, lipids, oxidative stress and inflammatory biomarkers as TNF-α levels individuals with or without diabetes and NALFD [15, 16, 66, 67].

Our nutraceutical also contains the standardised fraction “Picroliv” of the root of *Picrorhiza kurroa* [20]. Picroliv can prevent lipid peroxidation, inhibit the production of reactive oxygen species, and neutralize free oxygen radicals [21]. Hepato-protective effects in humans have only been evaluated in one study. Our study thus confirms its positive effects on the liver [68].

All these previous results confirm that a nutraceutical containing curcumin, ω-3 PUFAs, BPF, artichoke leaf extract, black seed oil, pricoliv, GHS, SAMe and other natural ingredients reduce CAP score (by ~ 12%).

Based on these evidences, the synergic effect of all these bioactive components with antioxidant properties would represent a novel approach to treat NAFLD. Although baseline total cholesterol and triglyceride levels were statistically higher in the treatment group than in placebo, no statistically significant difference in the change in these lipids was observed during the study. Both groups reached the same variation in lipids. This finding suggests that the effects of the nutraceutical on the liver are not mediated by changes in blood lipids. The hepatic steatosis is a clinical condition not always associated with a change in transaminases. Therefore, we do not always expect a reduction in transaminases following a treatment for hepatic steatosis [69, 70].

Two previous studies [15, 71] showed that 12 weeks of treatments with nutraceuticals did not have any beneficial effect on the metabolic features of NAFLD. However, in line with our findings, the nutraceuticals reduced the hepatic manifestation of it.

Subgroup analysis showed that the CAP score reduction was even greater in those with aged 60 or less, low baseline HDL-C, with AST reduction as well as in men. The result obtained in men is plausible because gender difference exists in metabolic, inflammatory, oxidative status and hormonal pathways [72, 73]. A different sex-dependent expression of genes of hepatic metabolism that are involved in the accumulation of triglycerides exists [74]. These differences may contribute not only to the different prevalence of NAFLD between genders [72, 73] but also to the response to the treatments.

The higher effect of the nutraceutical on subjects younger than 60 with hepatic steatosis is not surprising, because the aging is characterized by progressive physiologic changes, that influence both pharmacokinetics and pharmacodynamics of drugs in elderly patients [75]. These changes in older subjects have been associated with both reduced effectiveness and increased risk of adverse drug reactions to different drugs compared to younger subjects [75]. It is conceivable that the changes in pharmacokinetics and pharmacodynamics that are linked to the age-related drugs response may also influence the results of our study. However, further studies are needed to corroborate this hypothesis.

In addition, we found a greater reduction in liver fat content and an improvement in the degree of steatosis especially in subjects with low baseline HDL-C. This result is quite interesting. It was demonstrated that HDL-C inhibits the activation of SREBP-1 and decreased the expression levels of SREBP-1 target genes, probably by increasing levels of cellular cholesterol, suggesting that the maintain of serum HDL-C levels may be important to prevent abnormal lipid synthesis and the fat accumulation in liver [76]. Low HDL-C is also a component of the MS spectrum that is associated with NAFLD. Therefore, it is plausible that subjects with low HDL-C benefits more than other from taking the present nutraceutical. Further studies are needed to explain these mechanisms.

In our study there was a lack of the anti-inflammatory effects of the nutraceutical as evidenced by the fact that the change in concentration of IL-1β, IL-6 and TNF-α did not differ between the groups after 12 weeks of intervention (Additional file 4: Table S4). It has been demonstrated that the components of the nutraceutical (as SAMe, curcumin, BPF, TQ, PUFAs) improved NAFLD through other mechanisms as autophagy and stimulation of mitochondrial β-oxidation [19, 77–82].

However, the improvement of liver steatosis was not seen in patients with mild and moderate disease, but only in individuals with severe steatosis grade. This finding is not surprising. As shown in several clinical and preclinical studies, some nutraceuticals in NAFLD exert their beneficial effects in the early stage (due to the anti-inflammatory, antioxidant therapeutic properties) while others acts in the late stage of the disease (due to the antifibrotic properties, especially in individuals with several metabolic conditions) [71, 83, 84].

A short duration of the study also would limit the response to those with a severe disease [85].

Finally, it is plausible that the genetic background surrounding NAFLD influence the response to NALFD therapy. The lack of data concerning their effect on the therapeutic outcome remains an opened question.

Although, participants in the active group at baseline had a worse oxidative stress status (as suggested by BAP values) than controls, only these participants improved at the end of the study (Table 3). This result confirms that Livogen Plus® counteracts the mechanisms underlying the onset of hepatic steatosis.

This study had some limitations. First, the reduction in the CAP score is apparently lower than expected. In sample size calculation we referred to a less severe population [53]. However, the main finding is that individuals with severe hepatic steatosis at baseline have the greatest benefit from the present nutraceutical compared to those with mild/moderate hepatic steatosis. Second, it is well recognised liver biopsy is the only sensitive and reliable procedure for the diagnosis of liver steatosis [86]. However, it cannot be routinely because an invasive method [87]. In addition, this procedure may not always be representative if the underlying liver damage is not evenly distributed among the whole hepatic tissues [87]. Abdominal ultrasound is the first-line method but characterised by a low sensitivity when liver fat content is < 30% [86]. Other imaging techniques are costly and not affordable for all patients [86]. CAP score has both a good sensitivity and specificity in detecting the fatty liver [48, 86]. Third, the duration of the present study may not accurately capture the long-term effects of a nutraceutical on individuals with NAFLD [88].

However, there is the potential for increased participant attrition when study duration is increased; this may be due to illness, death or loss of interest in continued participation [89].

We chose thus 12 weeks of treatment based on previous studies on the treatment of NAFLD with nutraceuticals or dietary restrictions or medications [15, 90, 91].

Finally, a reduction in γGT is reported in placebo group but not in the treated group. Though the nutraceutical does not significantly reduce transaminases, so we do not expect a reduction in γGT in the treatment group. Moreover, it is well known that men have higher values of γGT than women [92, 93]. At baseline (n = 70/70), 56% (n = 39) in placebo and 50% (n = 35) in nutraceutical were males. As a consequence of the dropout, we lost 4 and 2 males in placebo and nutraceutical group respectively (54% vs. 53%). We may assume that in placebo γGT fell consequently.

Furthermore, γGT is abundant in liver, kidney, pancreas and intestine. As a screening test for liver disease, the γGT level has a very low specificity. Of course the results from the subgroup analysis should be interpreted with caution as they are only used for the generation of hypotheses for future studies. Despite these limitations, this study has important strengths. Several nutraceuticals are available on the market to treat NAFLD. However, not all of them have been tested in proper clinical trials [94]. Among the nutraceuticals currently available, the present product (Livogen Plus®) contains the largest number of antioxidants species already tested alone and, now, in combination in the present RCT. Another strength is that we considered all the possible confounding factors to confirm the efficacy of the nutraceutical on NAFLD*.*

## Conclusion

A new nutraceuticals combination significantly reduced the liver fat content in 62% of participants with severe NAFLD compared to 37% in the placebo group. The CAP score reduction was greater in those with aged 60 or less, low baseline HDL-C, AST reduction as well as in men. Future studies are needed to assess whether long-term supplementation with this nutraceutical can reduce the severity of NAFLD.

## Supplementary Information

**Additional file 1: Table S1.** Dietary advice, servings of various food categories consumed daily, weekly, or monthly during the study according to the treatments.

**Additional file 2: Table S2.** Nutrient profile of the overall diet according to the treatments.

**Additional file 3: Table S3.** Nutrients intake assessment and dietary intake changes during the study (intention to treat analysis).

**Additional file 4: Table S4.** Changes in clinical parameters at follow-up according to the treatments (intention to treat analysis).

**Additional file 5: Table S5.** Baseline and follow-up clinical characteristics of participants according to the treatments (per-protocol analysis).

**Additional file 6: Table S6.** Nutrients intake assessment and dietary intake changes during the study (per-protocol analysis).

**Additional file 7: Table S7.** Changes in clinical parameters at follow-up according to the treatments (per-protocol analysis).

**Additional file 8: Figure S1.** Prevalence of improvement of Liver Steatosis grade according to the treatments and severity of hepatic steatosis at baseline (per-protocol analysis). Prevalence between groups by Chi-square test with adjustment by General Linear Model.

**Additional file 9: Figure S2.** Prevalence of improvement of liver steatosis grade in the subgroups according to the treatments (per-protocol analysis). Prevalence between groups by Chi-square test with adjustment by General Linear Model.

**Additional file 10: Figure S3.** Livogen and placebo does not increases viability of McA-Rh-7777 cells. Semi-confluent cultures of rat hepatoma cell line (McA Rh-7777) incubated with nutraceutical and placebo (12.5 and 25 µg/mL) for 24 h. (A) Cell viability determined by MTT assay. Data are represented as mean ± SD. Abbreviations: MTT assay, 3-(4,5-dimethylthiazol-2-yl)-2,5-diphenyltetrazolium bromide assay; SD, standard deviation.

**Additional file 11: Table S8.** Antioxidant activity of nutraceutical and placebo extract.

**Additional file 12: Table S9.** Safety assessment of Livogen.

## Data Availability

The datasets used and/or analysed during the current study are available from the corresponding author on reasonable request.

## Abbreviations

NAFLD: Non-alcoholic fatty liver disease
CAP: Controlled attenuation parameter
VCTE: Vibration-controlled transient elastography
IR: Insulin resistance
T2D: Type 2 diabetes
NASH: Non-alcoholic steatohepatitis
RCT: Randomized controlled trial
ITT analysis: Intention-to-treat analysis
PP: Per-protocol analysis
BMI: Body mass index
WHR: Waist to hip ratio
FM: Fat mass
SBP: Systolic blood pressure
DBP: Diastolic blood pressure
HOMA-IR: Homeostatic model assessment of insulin resistance
AST: Aspartate aminotransferase
ALT: Alanine aminotransferase
γGT: Gamma glutamyltransferase
TC: Total cholesterol
TG: Triglycerides
HDL-C: High density lipoprotein cholesterol
BAP: Biological antioxidant potential
IL-1β: Interleukin-1β
IL-6: Interleukin-6
TNF-α: Tumor necrosis factor α
