# Supplementary material for: A new nutraceutical (Livogen Plus®) improves liver steatosis in adults with non-alcoholic fatty liver disease
Source: J Transl Med. 2022 Aug 19;20:377. doi: 10.1186/s12967-022-03579-1 (PMC9392294; doi:10.1186/s12967-022-03579-1)
Supplement: Supplementary file 1 — Additional file 1: Table S1. Dietary advice, servings of various food categories consumed daily, weekly, or monthly during the study according to the treatments. [file 12967_2022_3579_MOESM1_ESM.docx]

| **Table S1** Dietary advice, servings of various food categories consumed daily, weekly or monthly during the study according to the treatments | | |
| --- | --- | --- |
| Food categories | **Placebo** | **Nutraceutical** |
| Whole-grain products, serving/day | 4-5 | 4-5 |
| Legumes, serving/wk | 3 | 3 |
| Fruit/vegetables, serving/day | 8-10 | 8-10 |
| Milk and yogurt, serving/day | 1-2 | 1-2 |
| Cheese, serving/wk | <3 | <3 |
| Poultry, serving/wk | <3 | <3 |
| Fish, serving/wk | ≥3 | ≥3 |
| Red meat, serving/wk | <2 | <2 |
| Eggs, serving/month | 2 | 2 |
| Extravirgin olive oil, g/day | 20-30 | 20-30 |
| Sweets serving/wk | 1 | 1 |
| ***Note.*** | | |
