# Supplementary material for: A new nutraceutical (Livogen Plus®) improves liver steatosis in adults with non-alcoholic fatty liver disease
Source: J Transl Med. 2022 Aug 19;20:377. doi: 10.1186/s12967-022-03579-1 (PMC9392294; doi:10.1186/s12967-022-03579-1)
Supplement: Supplementary file 2 — Additional file 2: Table S2. Nutrient profile of the overall diet according to the treatments. [file 12967_2022_3579_MOESM2_ESM.docx]

| **Table S2** Nutrient profile of the overall diet according to the treatments | | | |
| --- | --- | --- | --- |
| Variables | **Placebo (n=70)** | **Nutraceutical (n=70)** | *p-value* |
| Calories Intake (Kcal) | 2045±443 | 2020±451 | 0.73 |
| Carbohydrates (%) | 50±8 | 48±8 | 0.18 |
| Proteins (%) | 14±3 | 15±3 | 0.30 |
| Animal protein (g) | 45±19 | 47±18 | 0.50 |
| Vegetable protein (g) | 29±9 | 27±9 | 0.25 |
| Fats (%) | 36±7 | 37±7 | 0.11 |
| Alcohol (g) | 6.1±7.8 | 4.4±6.4 | 0.15 |
| Cholesterol (g) | 219±97 | 235±84 | 0.29 |
| ***Note***. Difference between means by unpaired samples t test | | | |
