# Supplementary material for: A new nutraceutical (Livogen Plus®) improves liver steatosis in adults with non-alcoholic fatty liver disease
Source: J Transl Med. 2022 Aug 19;20:377. doi: 10.1186/s12967-022-03579-1 (PMC9392294; doi:10.1186/s12967-022-03579-1)
Supplement: Supplementary file 3 — Additional file 3: Table S3. Nutrients intake assessment and dietary intake changes during the study (intention to treat analysis). [file 12967_2022_3579_MOESM3_ESM.docx]

| **Table S3** Nutrients intake assessment and dietary intake changes during the study (Intention To Treat analysis) | | | | | | | |
| --- | --- | --- | --- | --- | --- | --- | --- |
|  | ***Baseline*** | | | ***Dietary Changes*** | | | |
| Variables | **Placebo**  **(n=65)** | **Nutraceutical (n=62)** | *p-value* | **Placebo (n=65)** | **Nutraceutical (n=62)** | | *p-value* |
| Calories Intake (Kcal) | 2004±403 | 2032±469 | 0.71 | -269±392 | | -261±447 | 0.91 |
| Carbohydrates (%) | 50±8 | 48±8 | 0.13 | -6±13 | | -1.4±15 | 0.07 |
| Proteins (%) | 14±2 | 15±2 | 0.09 | -0.4±4 | | -0.9±3 | 0.43 |
| Animal protein (g) | 42±16 | 45±16 | 0.25 | -1.1±17 | | -7.0±16 | 0.045 |
| Vegetable protein (g) | 28±8 | 28±9 | 0.86 | -2.1±8 | | 1.6±9 | 0.014 |
| Fats (%) | 36±7 | 37±7 | 0.12 | -4.2±10 | | -7.3±9 | 0.07 |
| Alcohol (g) | 5.6±7 | 4.3±6 | 0.28 | -2.7±5 | | -2.0±4 | 0.36 |
| Cholesterol (g) | 210±89 | 238±81 | 0.06 | -27.6±94 | | -61.5±80 | 0.030 |
| ***Note.*** Difference between means by unpaired samples t test | | | | | | | |
