# Supplementary material for: A new nutraceutical (Livogen Plus®) improves liver steatosis in adults with non-alcoholic fatty liver disease
Source: J Transl Med. 2022 Aug 19;20:377. doi: 10.1186/s12967-022-03579-1 (PMC9392294; doi:10.1186/s12967-022-03579-1)
Supplement: Supplementary file 4 — Additional file 4: Table S4. Changes in clinical parameters at follow-up according to the treatments (intention to treat analysis). [file 12967_2022_3579_MOESM4_ESM.docx]

| **Table S4** Changes in clinical parameters at follow-up according to the treatments (Intention To Treat analysis) | | | |
| --- | --- | --- | --- |
| **Variables** | **Placebo**  **(n=65)** | **Nutraceutical**  **(n=62)** | ***p-value*** |
| Follow-up duration (days) | 86±4 | 85±3 | 0.15 |
| Adherence to treatment (≥ 80 %, %) | 83 | 89 | 0.44 |
| Weight (Kg) | -0.8±3 | -1.1±2 | 0.48 |
| BMI (Kg/m^2^) | -0.35±1.0 | -0.40±0.8 | 0.76 |
| WHR | -0.02±0.04 | -0.03±0.05 | 0.23 |
| FM (kg) | -0.07±2 | 0.17±2 | 0.53 |
| CAP score (dB/m) | -25±41  - | -29±38 | 0.54 |
| Stiffness (kPa) | 0.1±1 | -0.2±1 | 0.13 |
| Glucose (mg/dL) | 1.4±8 | -0.7±7 | 0.12 |
| Insulin (mU/L) | -1±8  - | 2±6 | 0.67 |
| HOMA-IR | -0.2±2 | -0.1±3 | 0.79 |
| TC (mg/dL) | 2±23 | 6±30 | 0.42 |
| TG (mg/dL) | 6±44 | -9±65 | 0.15 |
| HDL-C (mg/dL) | 3±6 | 2±5 | 0.46 |
| Albumin (g/dl) | 0.07±0.3 | 0.09±0.6 | 0.83 |
| AST (IU/L) | -2±10 | -1±8 | 0.45 |
| ALT (IU/L) | -1±17 | -0.3±13 | 0.75 |
| γGT (UI/L) | -2.5±8 | 0.6±7 | 0.026 |
| Creatinine (mg/dL) | 0.02±0.1 | -0.004±0.1 | 0.12 |
| CRP (mg/L) | -0.1±1 | -0.4±3 | 0.54 |
| BAP (μmol/L) | 171±647 | 117±460 | 0.71 |
| ***Cytokine evaluation*** | | | |
| IL-1β (pg/mL) | -1.9±12 | -2.1±4 | 0.18 |
| IL-6 (pg/mL) | -2.3±7 | -1.3±2 | 0.57 |
| TNF-α (pg/mL) | 0.2±10 | -3.4±24 | 0.96 |
| ***Note.*** BMI = body mass index, WHR = waist to hip ratio, FM = fat mass, CAP = controlled attenuation parameter, HOMA-IR = homeostatic model assessment of insulin resistance, TC = total cholesterol, TG = triglycerides, HDL-C = high density lipoprotein cholesterol, AST = aspartate aminotransferase, ALT = alanine aminotransferase, γGT = gamma glutamyltransferase, BAP = biological antioxidant potential, IL-1β = interleukin-1β, IL-6 = interleukin-6, TNF-α = tumor necrosis factor α. Difference between means by unpaired samples t test; differences in BAP, IL-1β, IL-6 and TNF α by Mann-Whitney U test | | | |
