# Supplementary material for: A new nutraceutical (Livogen Plus®) improves liver steatosis in adults with non-alcoholic fatty liver disease
Source: J Transl Med. 2022 Aug 19;20:377. doi: 10.1186/s12967-022-03579-1 (PMC9392294; doi:10.1186/s12967-022-03579-1)
Supplement: Supplementary file 5 — Additional file 5: Table S5. Baseline and follow-up clinical characteristics of participants according to the treatments (per-protocol analysis). [file 12967_2022_3579_MOESM5_ESM.docx]

| **Table S5** Baseline and follow-up clinical characteristics of participants according to the treatments (Per-Protocol analysis i.e. in those with adherence ≥ 80% ) | | | | | | | |
| --- | --- | --- | --- | --- | --- | --- | --- |
|  | **Placebo**  **(n=54)** | | | **Nutraceutical**  **(n=55)** | | |  |
| Variables | *Basal* | *Follow-up* | *p-value*  *(paired t test)* | *Basal* | *Follow-up* | *p-value*  *(paired t test)* | *p-value*  *(unpaired t test between basal values )* |
| Weight (Kg) | 80±11 | 79±11 | 0.012 | 77±12 | 76±12 | <0.001 | 0.20 |
| BMI (Kg/m^2^) | 29.8±3 | 29.4±3 | 0.006 | 28.6±4 | 28.2±4 | <0.001 | 0.09 |
| WHR | 0.97±0.7 | 0.95±0.07 | 0.007 | 0.96±0.07 | 0.93±0.06 | <0.001 | 0.79 |
| FM (Kg) | 25.8±7 | 25.6±6 | 0.55 | 23.9±7 | 24.0±7 | 0.61 | 0.15 |
| SBP (mmHg) | 119±12 | 118±10 | 0.55 | 118±13 | 117±13 | 0.56 | 0.58 |
| DBP (mmHg) | 77±9 | 76±9 | 0.27 | 77±9 | 74±9 | 0.011 | 0.81 |
| CAP score (dB/m) | 304±32 | 280±42 | <0.001 | 297±32 | 266±42 | <0.001 | 0.26 |
| *a*CAP score (dB/m)* | 306±4 | / | / | 296±4 | / | / | 0.11 |
| Stiffness (kPa) | 4.6±1.1 | 4.8±1.2 | 0.37 | 4.7±1.0 | 4.6±1.1 | 0.23 | 0.65 |
| Glucose (mg/dL) | 89±9 | 91±9 | 0.19 | 92±10 | 91±9 | 0.26 | 0.12 |
| Insulin (mU/L) | 14±11 | 13±8 | 0.32 | 13±7 | 11±6 | 0.023 | 0.38 |
| HOMA-IR | 3.2±2.5 | 3.0±1.8 | 0.40 | 3.0±1.8 | 2.5±1.4 | 0.021 | 0.47 |
| TC (mg/dL) | 191±35 | 191±32 | 0.79 | 204±31 | 209±40 | 0.20 | 0.035 |
| TG (mg/dL) | 117±51 | 122±60 | 0.44 | 150±87 | 136±96 | 0.13 | 0.018 |
| HDL-C (mg/dL) | 49±11 | 52±12 | 0.003 | 49±10 | 51±11 | 0.013 | 0.97 |
| Albumin (g/dl) | 4.4±0.3 | 4.5±0.4 | 0.20 | 4.4±0.5 | 4.5±0.3 | 0.23 | 0.73 |
| AST (IU/L) | 22±13 | 20±11 | 0.09 | 21±6 | 21±6 | 0.67 | 0.52 |
| ALT (IU/L) | 27±25 | 25±20 | 0.49 | 23±13 | 23±12 | 0.95 | 0.36 |
| γGT (UI/L) | 27±19 | 25±18 | 0.004 | 24±13 | 24±14 | 0.49 | 0.23 |
| Creatinine (mg/dL) | 0.85±0.1 | 0.87±0.1 | 0.10 | 0.84±0.2 | 0.83±0.2 | 0.44 | 0.71 |
| CRP (mg/L) | 3.8±1.7 | 3.7±1.3 | 0.55 | 4.1±3.7 | 3.8±1.8 | 0.50 | 0.60 |
| BAP (μmol/L) | 1978±535 | 2175±395 | 0.06 | 1596±437 | 1725±501 | 0.06 | 0.001 |
| ***Cytokine evaluation*** | | | | | | | |
| IL-1β (pg/mL) | 16.6±5 | 15.4±12 | <0.001 | 15.9±3 | 13.7±3 | 0.002 | 0.88 |
| IL-6 (pg/mL) | 8.4±3 | 6.9±1 | 0.001 | 8.2±2 | 6.9±1 | 0.001 | 0.79 |
| TNF-α (pg/mL) | 12.9±9 | 12.4±8 | 0.52 | 16.1±25 | 12.4±5 | 0.69 | 0.58 |
| **a*CAP score adjusted for serum triglycerides at baseline.  ***Note.*** BMI = body mass index, WHR = waist to hip ratio, FM = fat mass, SBP = systolic blood pressure, DBP = diastolic blood pressure, CAP = controlled attenuation parameter, HOMA-IR = homeostatic model assessment of insulin resistance, TC = total cholesterol, TG = triglycerides, HDL-C = high density lipoprotein cholesterol, AST = aspartate aminotransferase, ALT = alanine aminotransferase, γGT = gamma glutamyltransferase, BAP = biological antioxidant potential, IL-1β = interleukin-1β, IL-6 = interleukin-6, TNF-α = tumor necrosis factor α. Difference between means by unpaired samples t test; within group variation by paired Student’s t test (two tailed); differences in BAP, IL-1β, IL-6 and TNF α by Mann-Whitney U test and Wilcoxon signed-rank test | | | | | | | |
