# Supplementary material for: A new nutraceutical (Livogen Plus®) improves liver steatosis in adults with non-alcoholic fatty liver disease
Source: J Transl Med. 2022 Aug 19;20:377. doi: 10.1186/s12967-022-03579-1 (PMC9392294; doi:10.1186/s12967-022-03579-1)
Supplement: Supplementary file 6 — Additional file 6: Table S6. Nutrients intake assessment and dietary intake changes during the study (per-protocol analysis). [file 12967_2022_3579_MOESM6_ESM.docx]

| **Table S6** Nutrients intake assessment and dietary intake changes during the study (Per-Protocol analysis) | | | | | | | |
| --- | --- | --- | --- | --- | --- | --- | --- |
|  | ***Baseline*** | | | ***Dietary Changes*** | | | |
| Variables | **Placebo**  **(n=54)** | **Nutraceutical (n=55)** | *p-value* | **Placebo (n=54)** | **Nutraceutical (n=55)** | | *p-value* |
| Calories Intake (Kcal) | 1967±417 | 2031±486 | 0.46 | -273±413 | -256±466 | | 0.84 |
| Carbohydrates (%) | 50±8 | 49±78 | 0.38 | -6.2±13 | | -1.4±16 | 0.09 |
| Proteins (%) | 14±2 | 14±2 | 0.25 | -0.3±4 | | -0.8±4 | 0.50 |
| Animal protein (g) | 42±16 | 45±16 | 0.25 | -1.3±18 | | -6.8±16 | 0.10 |
| Vegetable protein (g) | 28±8 | 28±9 | 0.86 | -1.8±8 | | 1.8±9 | **0.035** |
| Fats (%) | 36±7 | 37±7 | 0.28 | -4.1±11 | | -6.9±9 | 0.14 |
| Alcohol (g) | 5.8±8 | 4.1±6 | 0.19 | -2.8±5 | | -2.0±4 | 0.36 |
| Cholesterol (g) | 205±90 | 229±76 | 0.14 | -31±102 | | -60±77 | 0.09 |
| ***Note.*** Difference between means by unpaired samples t test | | | | | | | |
