# Supplementary material for: A new nutraceutical (Livogen Plus®) improves liver steatosis in adults with non-alcoholic fatty liver disease
Source: J Transl Med. 2022 Aug 19;20:377. doi: 10.1186/s12967-022-03579-1 (PMC9392294; doi:10.1186/s12967-022-03579-1)
Supplement: Supplementary file 7 — Additional file 7: Table S7. Changes in clinical parameters at follow-up according to the treatments (per-protocol analysis). [file 12967_2022_3579_MOESM7_ESM.docx]

| **Table S7** Changes in clinical parameters at follow-up according to the treatments (Per-Protocol analysis) | | | |
| --- | --- | --- | --- |
| **Variables** | **Placebo**  **(n=54)** | **Nutraceutical**  **(n=55)** | ***p-value*** |
| Follow-up duration (days) | 86±4 | 85±3 | 0.21 |
| Adherence to treatment (≥ 80 %, %) | 95±5 | 94±6 | 0.62 |
| Weight (Kg) | -0.9±3 | -1.2±2 | 0.52 |
| BMI (Kg/m^2^) | -0.4±1.0 | -0.4±0.8 | 0.82 |
| WHR | -0.01±0.04 | -0.03±0.05 | 0.09 |
| FM (kg) | -0.2±2 | 0.1±2 | 0.43 |
| CAP score (dB/m) | -24±42 | -31±39 | 0.34 |
| Stiffness (kPa) | 0.1±1 | -0.2±1 | 0.14 |
| Glucose (mg/dL) | 1.6±9 | -1.1±7 | 0.08 |
| Insulin (mU/L) | -1±9 | -2±6 | 0.63 |
| HOMA-IR | -0.2±2 | -0.1±3 | 0.82 |
| TC (mg/dL) | 0.8±23 | 5.4±31 | 0.39 |
| TG (mg/dL) | 5±47 | -14±66 | 0.09 |
| HDL-C (mg/dL) | 2.6±6 | 1.9±6 | 0.54 |
| Albumin (g/dl) | 0.08±0.4 | 0.1±0.6 | 0.80 |
| AST (IU/L) | -2±10 | -0.4±6 | 0.21 |
| ALT (IU/L) | -2±18 | 0.1±11 | 0.54 |
| γGT (UI/L) | -2±5 | 0.7±7 | 0.019 |
| Creatinine (mg/dL) | 0.02±0.1 | -0.01±0.1 | 0.10 |
| CRP (mg/L) | -0.1±2 | -0.3±3 | 0.72 |
| BAP (μmol/L) | 198±624 | 129±443 | 0.56 |
| ***Cytokine evaluation*** | | | |
| IL-1β (pg/mL) | -1.2±13 | -2.2±4 | 0.55 |
| IL-6 (pg/mL) | -1.5±3 | -1.4±3 | 0.97 |
| TNF-α (pg/mL) | -0.5±8 | -3.7±25 | 0.51 |
| ***Note.*** BMI = body mass index, WHR = waist to hip ratio, FM = fat mass, CAP = controlled attenuation parameter, HOMA-IR = homeostatic model assessment of insulin resistance, TC = total cholesterol, TG = triglycerides, HDL-C = high density lipoprotein cholesterol, AST = aspartate aminotransferase, ALT = alanine aminotransferase, γGT = gamma glutamyltransferase, BAP = biological antioxidant potential, IL-1β = interleukin-1β, IL-6 = interleukin-6, TNF-α = tumor necrosis factor α. Difference between means by unpaired samples t test; differences in BAP, IL-1β, IL-6 and TNF α by Mann-Whitney U test | | | |
