# Supplementary figures and images for: A new nutraceutical (Livogen Plus®) improves liver steatosis in adults with non-alcoholic fatty liver disease
Source: J Transl Med. 2022 Aug 19;20:377. doi: 10.1186/s12967-022-03579-1 (PMC9392294; doi:10.1186/s12967-022-03579-1)

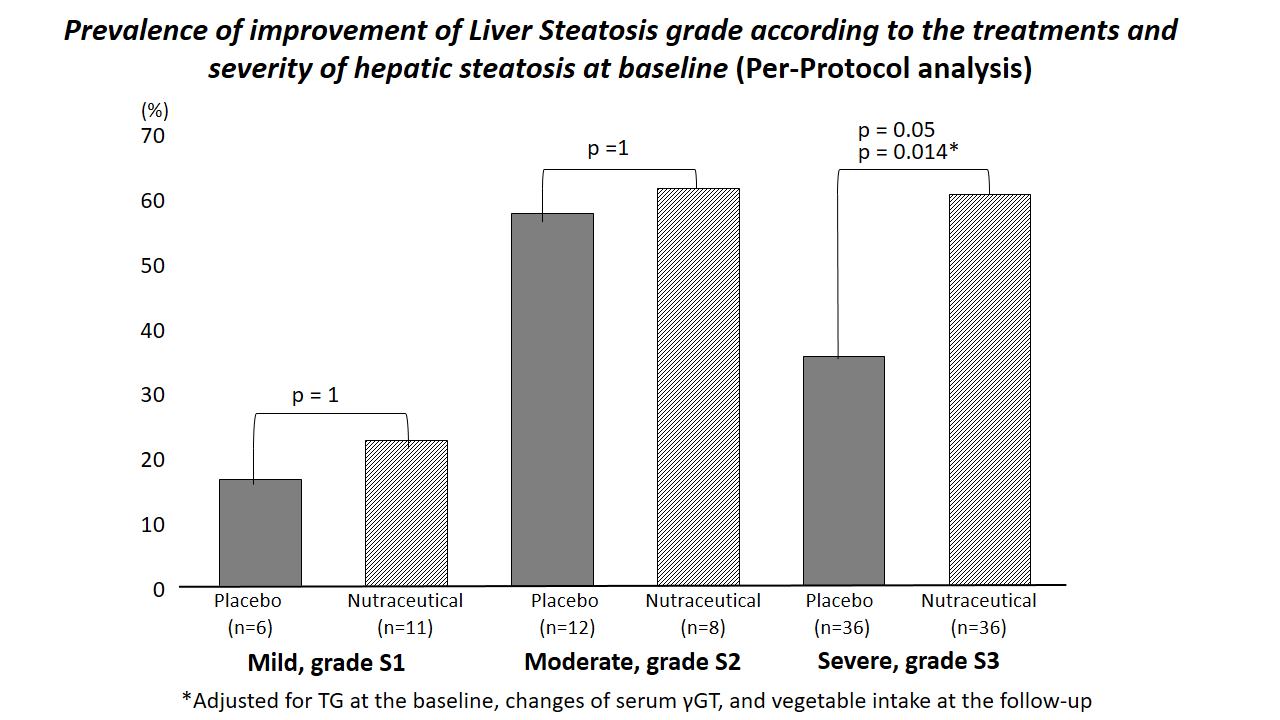

Supplement: Supplementary file 8 — Additional file 8: Figure S1. Prevalence of improvement of Liver Steatosis grade according to the treatments and severity of hepatic steatosis at baseline (per-protocol analysis). Prevalence between groups by Chi-square test with adjustment by General Linear Model. [file 12967_2022_3579_MOESM8_ESM.tif]

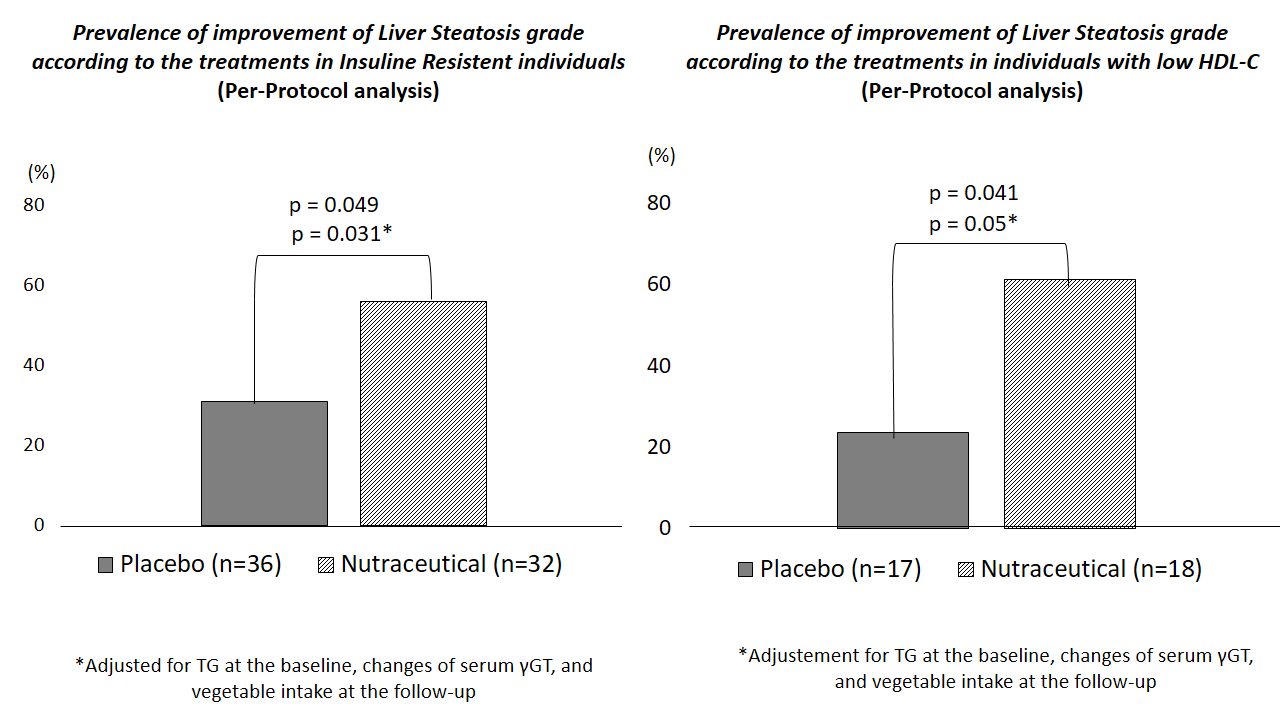

Supplement: Supplementary file 9 — Additional file 9: Figure S2. Prevalence of improvement of liver steatosis grade in the subgroups according to the treatments (per-protocol analysis). Prevalence between groups by Chi-square test with adjustment by General Linear Model. [file 12967_2022_3579_MOESM9_ESM.tif]

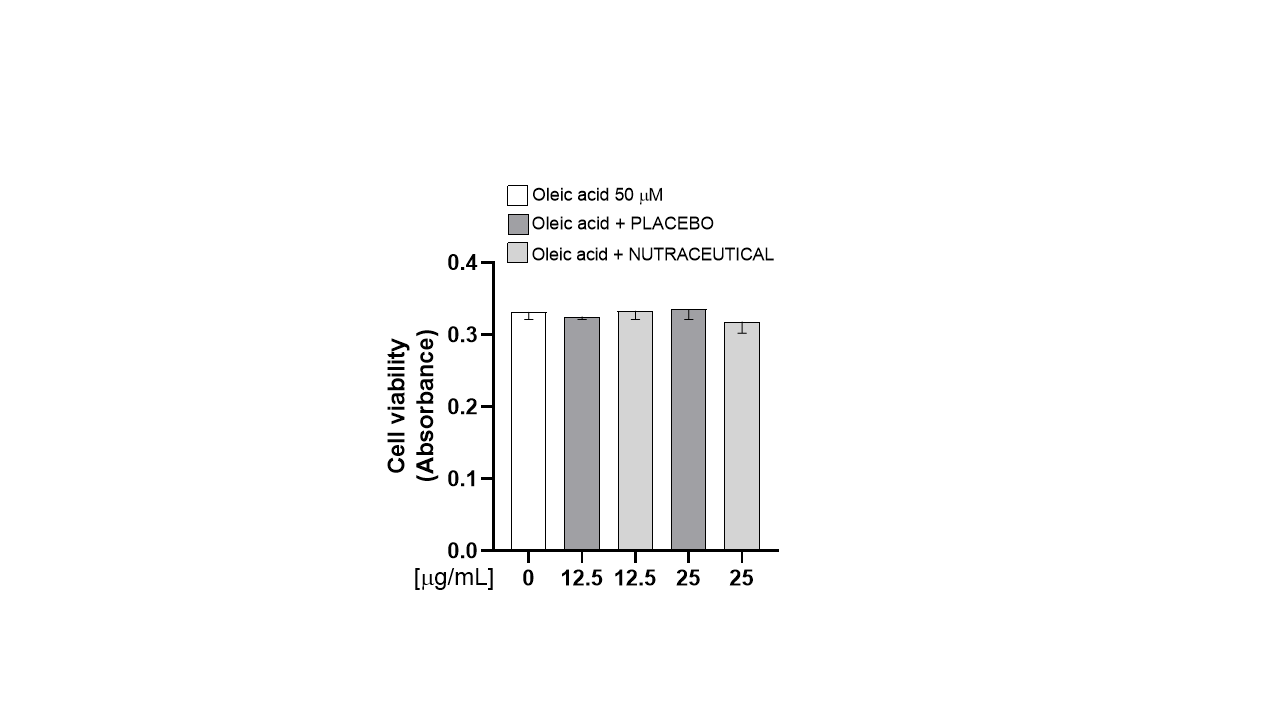

Supplement: Supplementary file 10 — Additional file 10: Figure S3. Livogen and placebo does not increases viability of McA-Rh-7777 cells. Semi-confluent cultures of rat hepatoma cell line (McA Rh-7777) incubated with nutraceutical and placebo (12.5 and 25 µg/mL) for 24 h. (A) Cell viability determined by MTT assay. Data are represented as mean ± SD. Abbreviations: MTT assay, 3-(4,5-dimethylthiazol-2-yl)-2,5-diphenyltetrazolium bromide assay; SD, standard deviation. [file 12967_2022_3579_MOESM10_ESM.tif]
