# Supplementary material for: A new nutraceutical (Livogen Plus®) improves liver steatosis in adults with non-alcoholic fatty liver disease
Source: J Transl Med. 2022 Aug 19;20:377. doi: 10.1186/s12967-022-03579-1 (PMC9392294; doi:10.1186/s12967-022-03579-1)
Supplement: Supplementary file 11 — Additional file 11: Table S8. Antioxidant activity of nutraceutical and placebo extract. [file 12967_2022_3579_MOESM11_ESM.docx]

| Table S8 Antioxidant activity of nutraceutical and placebo extract | | | |
| --- | --- | --- | --- |
| Variables | **Placebo** | **Nutraceutical** | ***p-value*** |
| Antioxidant activity (I %) | -3.6±0.21 | 82.9±0.36 | <0.001 |
| *Note.* I% = percentage of inhibition. Difference between means by unpaired samples t test | | | |
