# Supplementary material for: A new nutraceutical (Livogen Plus®) improves liver steatosis in adults with non-alcoholic fatty liver disease
Source: J Transl Med. 2022 Aug 19;20:377. doi: 10.1186/s12967-022-03579-1 (PMC9392294; doi:10.1186/s12967-022-03579-1)
Supplement: Supplementary file 12 — Additional file 12: Table S9. Safety assessment of Livogen. [file 12967_2022_3579_MOESM12_ESM.docx]

| **Table S9** Safety Assessment of Livogen | | |
| --- | --- | --- |
| **Ingredients** | **Maximum Safe Dosage** | **Safety Studies References** |
| Cavacurmin^®^ | Curcumin-cyclodextrin complex CAVACURMIN^®^ at a dosage of 2 grams per day showing no adverse effects. Curcumin in general has been shown well tolerated at high doses of up to 12 g/day. | Hundshammera C, Schönb C, Kimurac M, Furunec T, Teraoc K, Elgetia D, et al. Enhanced metabolic bioavailability of tetrahydrocurcumin after oral supplementation of a γ-cyclodextrin curcumin complex. Journal of Functional Foods. 2021; 79:104410.  Gupta SC, Patchva S, Koh W, Aggarwal BB. Discovery of curcumin, a component of golden spice, and its miraculous biological activities. Clin Exp Pharmacol Physiol. 2012;39:283-99.  Gupta SC, Patchva S, Aggarwal BB. Therapeutic roles of curcumin: lessons learned from clinical trials. AAPS J. 2013;15:195-218. |
| AlaskOmega^®^ | Dietary supplements providing 5 g/day EPA and DHA (Omega-3) are safe. | EFSA Panel on Dietetic Products NaA. Scientific opinion on the tolerable upper intake level of eicosapentaenoic acid (EPA), docosahexaenoic acid (DHA) and docosapentaenoic acid (DPA). EFSA Journal 2012;10:2815.  U.S. Food and Drug Administration. Qualified health claims: letters of enforcement discretionexternal link disclaimer. 2019. |
| Bergacyn^®^ | Bergacyn showed significant safety and efficacy in a clinical trial at 600 mg/day. | Musolino V, Gliozzi M, Bombardelli E, Nucera S, Carresi C, Maiuolo J, et al. The synergistic effect of Citrus bergamia and Cynara cardunculus extracts on vascular inflammation and oxidative stress in non-alcoholic fatty liver disease. J Tradit Complement Med. 2020;10:268-274. |
| Black Seed Oil-ThymoQuin™ (*Nigella Sativa*) | Recent meta-analysis shows that black seed supplementation (1000-2000 mg/day from 8 weeks to 12 weeks) is well tolerated without any adverse effects among NAFLD patients. | Tang G, Zhang L, Tao J, Wei Z. Effect of Nigella sativa in the treatment of nonalcoholic fatty liver disease: A systematic review and meta-analysis of randomized controlled trials. Phytother Res. 2021;35:4183-4193. |
| Picroliv^®^ (*Picrorhiza Kurroa*) root | Picrorhiza dosage ranges from 400 mg to 1,500 mg daily. | Girish C, Pradhan SC. Drug development for liver diseases: focus on picroliv, ellagic acid and curcumin. Fundam Clin Pharmacol. 2008;22:623-632.  Hechtman L. Clinical naturopathic medicine. Elsevier Health Sciences, 2018. |
| Opitac^™^ (reduced Glutathione) | OPITAC™ Glutathione is the world’s only glutathione that has been notified to the U.S. Food and Drug Administration (FDA) as GRAS (the indication of safety as a food ingredient). Recommended dosage up to 500 mg/day. | Allen J, Bradley RD. Effects of oral glutathione supplementation on systemic oxidative stress biomarkers in human volunteers. J Altern Complement Med. 2011;17:827-833.21875351  Available online:  https://opitacglutathione.com/faq/ |
| SAMe (as S-Adenosyl-L-Methionine) | Safety assessed clinically up to 1200 mg/day. | Guo T, Chang L, Xiao Y, Liu Q. S-adenosyl-L-methionine for the treatment of chronic liver disease: a systematic review and meta-analysis. PLoS One. 2015;10:e0122124. |
| Artichoke Leaf Extract | A clinical trial have demonstrated that Artichoke Leaf Extract is safe and well tolerated (up to 2700 mg/day for two months in patients suffering NASH). | Rangboo V, Noroozi M, Zavoshy R, Rezadoost SA, Mohammadpoorasl A. The Effect of Artichoke Leaf Extract on Alanine Aminotransferase and Aspartate Aminotransferase in the Patients with Nonalcoholic Steatohepatitis. Int J Hepatol. 2016;2016:4030476. |
| Indole-3-Carbinol | In human studies, doses were generally between 200 to 400 mg per day. | Hendler SS, Rorvik DM. PDR for Nutritional Supplements. 2nd ed: Thomson Reuters; 2008.  Available online: https://lpi.oregonstate.edu/mic/dietary-factors/phytochemicals/indole-3-carbinol |
| Silybin Phospholipids | Silybin phospholipids are safe and well tolerated without adverse effects (942 mg highest dose used in patients with liver damage). | Loguercio C, Festi D. Silybin and the liver: from basic research to clinical practice. World J Gastroenterol. 2011;17:2288-2301. |
| Milk Thistle | Safe and well tolerated among humans (up to 2100 mg/daily). | Soleimani V, Delghandi PS, Moallem SA, Karimi G. Safety and toxicity of silymarin, the major constituent of milk thistle extract: An updated review. Phytother Res. 2019;33:1627-1638.  Karimi G, Vahabzadeh M, Lari P, Rashedinia M, Moshiri M. "Silymarin", a promising pharmacological agent for treatment of diseases. Iran J Basic Med Sci. 2011;14:308-317.  Available online: https://www.nccih.nih.gov/health/milk-thistle |
| Holy Basil (*Ocimum sanctum Linn*.) | Holy Basil has been studied in a number of trials for safety and efficacy. A trial reported that 16 obese individuals supplemented with *Ocimum sanctum Linn.* 250 mg experienced occassional nausea. | Jamshidi N, Cohen MM. The Clinical Efficacy and Safety of Tulsi in Humans: A Systematic Review of the Literature. Evid Based Complement Alternat Med. 2017;2017:9217567.  Satapathy S, Das N, Bandyopadhyay D, Mahapatra SC, Sahu DS, Meda M. Effect of Tulsi (Ocimum sanctum Linn.) Supplementation on Metabolic Parameters and Liver Enzymes in Young Overweight and Obese Subjects. Indian J Clin Biochem. 2017;32:357-363. |
| Luteolin/quercetin | Safe and well tolerated among humans (up to 1000 mg/day). | Sotiropoulou M, Katsaros I, Vailas M, Lidoriki I, Papatheodoridis GV, Kostomitsopoulos NG, et al. Nonalcoholic fatty liver disease: The role of quercetin and its therapeutic implications. Saudi J Gastroenterol. 2021;27:319-330. |
| *Schisandra chinesis* | *Schisandra* is generally a well-tolerated up to 2000 mg/day standardized extract (1.3% lignan). | Available online:  https://botanicalinstitute.org/schisandra/,  Nowak A, Zakłos-Szyda M, Błasiak J, Nowak A, Zhang Z, Zhang B. Potential of *Schisandra chinensis* (Turcz.) Baill. in Human Health and Nutrition: A Review of Current Knowledge and Therapeutic Perspectives. Nutrients. 2019;11:333. |
| Mixed Tocotrienols Complex | No reported side effects. A risk of bleeding exists with doses greater than 1000 mg/day. | Owen KN, Dewald O. Vitamin E Toxicity. 2022 May 2. In: StatPearls [Internet]. Treasure Island (FL): StatPearls Publishing; 2022 Jan. |
| Livinol^®^ (*Garcinia*) | Toxicology studies showed no toxicity or deaths in animals given dosages of hydroxycitric acid (HCA) 5,000 mg/kg, equivalent to HCA 350 g in humans, or 233 times the maximum recommended human dosage of HCA 1.5 g/day. | Andueza N, Giner RM, Portillo MP. Risks Associated with the Use of Garcinia as a Nutritional Complement to Lose Weight. Nutrients. 2021;13:450.  Panda V, Ashar H, Srinath S. Antioxidant and hepatoprotective effect of Garcinia indica fruit rind in ethanol-induced hepatic damage in rodents. Interdiscip Toxicol. 2012;5:207-213. |
| Dandelion Root PE (4:1) (*Taraxacum*- officinale) | The use of dandelion in the amounts commonly found in food is generally considered safe (up to 50 g or more/day). | Available online:  https://www.nccih.nih.gov/health/dandelion, |
| Astazine^®^ (Natural Astaxanthin) | No applicable safety concerns for natural astaxanthin supplementation at 12 mg/day. | Brendler T, Williamson EM. Astaxanthin: How much is too much? A safety review. Phytother Res. 2019;33:3090-3111. |
